# A Fluorescent Probe to Measure DNA Damage and Repair

**DOI:** 10.1371/journal.pone.0131330

**Published:** 2015-08-26

**Authors:** Allison G. Condie, Yan Yan, Stanton L. Gerson, Yanming Wang

**Affiliations:** 1 Department of Radiology, Chemistry, and Biomedical Engineering, Case Western Reserve University, Cleveland, Ohio, United States of America; 2 Department of Pharmacology, Case Western Reserve University, Cleveland, OH, United States of America; 3 Department of Hematology and Oncology, Case Comprehensive Cancer Center, Case Western Reserve University, Cleveland, OH, United States of America; University of South Alabama Mitchell Cancer Institute, UNITED STATES

## Abstract

DNA damage and repair is a fundamental process that plays an important role in cancer treatment. Base excision repair (BER) is a major repair pathway that often leads to drug resistance in DNA-targeted cancer chemotherapy. In order to measure BER, we have developed a near infrared (NIR) fluorescent probe. This probe binds to a key intermediate, termed apurinic/apyrimidinic (AP) site, in the BER pathway where DNA damage and repair occurs. We have developed an assay to show the efficacy of the probe binding to AP sites and have shown that it can distinguish AP sites in DNA extract from chemotherapy treated cells. This probe has potential application in monitoring patient response to chemotherapy and evaluating new drugs in development.

## Introduction

As cancer continues to be a public health concern, tremendous efforts are being directed toward the development of new chemotherapies, a popular strategy for cancer treatment since Sidney Farber first introduced the antimetabolite aminopterin for use against leukemia [1]. Like aminopterin, many chemotherapeutic agents induce DNA damage ultimately leading to cell death [2]. Under normal physiological conditions, multiple pathways exist for the repair of damaged DNA. However, such pathways can subvert the effects of chemotherapy targeting DNA in cancer cells and trigger drug resistance. These repair pathways include homologous recombination, non-homologous end joining, nucleotide excision repair, mismatch repair, and base excision repair (BER) [3,4,5,6]. While all these pathways repair DNA damage, the types of damage they repair vary, with BER being particularly relevant to exogenous chemical damage such as that caused by chemotherapy [7,8,9].

**Fig 1:**
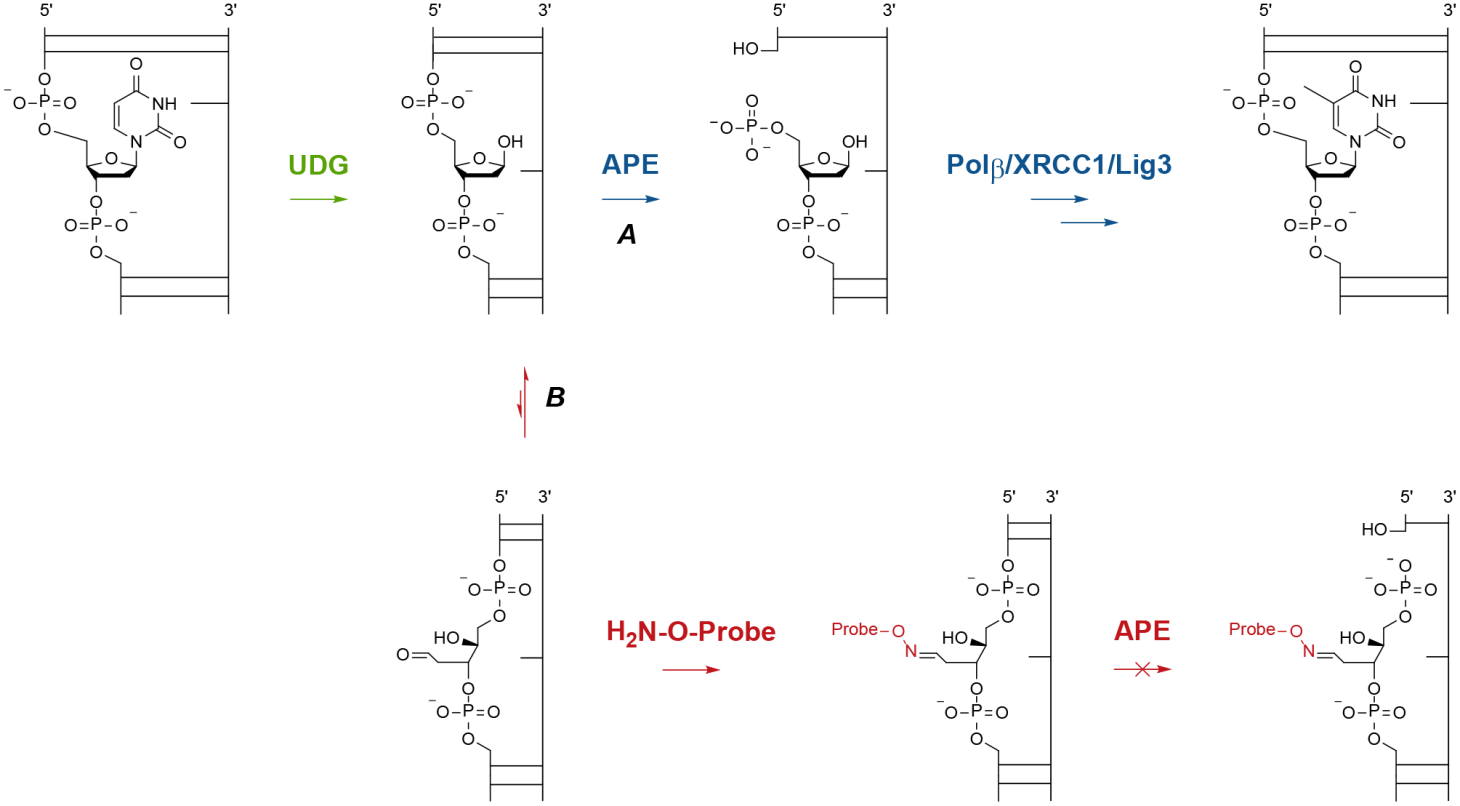
Molecular view of normal and interrupted BER. Path A: the short patch BER pathway following UDG removal of uracil in DNA. Path B: the proposed mechanism for interception of the reactive aldehyde present in the AP site with an aminooxy-tagged probe, which blocks further repair by APE and prevents the single strand break (SSB).

Damage to DNA can result from direct chemical modification of nucleotides or from accumulation of aberrant bases, such as uracil [10]. Regardless of the type of damage, the first step in the BER pathway is the excision of the damaged base by a glycosylase, which leaves the free ribose sugar termed abasic or AP (apurinic/apyrimidinic) site [6]. For example, uracil DNA glycosylase (UDG) [6,11,12,13] rapidly excises uracil from DNA to initiate the repair sequence (Fig 1). AP sites are the most common lesions in DNA, and if left unrepaired, can be mutagenic [14,15]. AP sites are formed following oxidative damage of DNA by reactive oxygen species (ROS) [16,17] and this oxidative damage is associated with cancer, heart disease, Parkinson disease, and aging [18,19]. Therefore, tools that detect and quantify AP sites are of broad interest to the medical and scientific communities.

Several research groups have developed tools to detect AP sites based on methods including fluorescence [20,21], nanopore ion detection [22], mass spectrometry [23,24], atomic force microscopy [25], electrochemistry [26], and electron paramagnetic resonance [27]. Some of these techniques employ a variety of molecular probes targeted to the AP site through various chemical features of the lesions [28]. Several probes containing an aminooxy moiety have been developed that covalently bind to the AP site aldehyde and form an oxime ether [29,30]. Among them, aldehyde reactive probe (ARP), based on biotin tethered to an alkoxyamine, detects AP sites in a colorimetric streptavidin-horseradish peroxidase in vitro assay [31]. Likewise, our research group has examined methoxyamine (MX) [32,33] and has pioneered in vivo imaging of BER by radiolabelling MX with carbon-11 for use with positron emission tomography (PET) in clinical research [30].

Previously developed aldehyde-reactive probe (ARP) and similar compounds that fluoresce in the UV-visible range are commonly used for in vitro detection and quantification of AP sites. Such assays have several drawbacks in studying DNA-targeted chemotherapies: 1) they are often limited to the study of AP sites in DNA of circulating cells in plasma induced by chemotherapeutic agents, which is only an indirect measure of AP site formation; 2) for direct detection and quantification of AP sites in tumor regions, dissection and homogenization of tumor tissues are required at each time point after therapeutic treatments, which make longitudinal studies impossible; 3) these assays are not readily repeatable due to sophisticated procedures. To overcome these drawbacks, we set out to develop novel probes with near-infrared fluorescence as represented by title compound **7**. NIR is superior to UV-visible light due to its deeper tissue penetration and less tissue scattering. It thus has the potential to be used for longitudinal imaging in live animals.

For preclinical studies, near infrared (NIR) fluorescence imaging has been a popular modality that is capable of longitudinal studies in animal models. Compared to PET, NIR imaging is cost-efficient, easy to operate, and has the advantage of multichannel imaging. Further, heptamethine cyanine dyes have been used extensively in NIR fluorescent imaging as contrast agents for tumor imaging [34]. For these reasons, we developed a cyanine-based NIR probe, compound **7**, that exhibits promising properties of binding to AP sites for detection and quantitation of DNA damage. Herein we report the design and synthesis of compound **7** and evaluation of its physicochemical and binding properties for direct detection and quantification of AP sites.

## Results and Discussion

### Chemistry

#### Synthesis of 7

**Fig 2:**
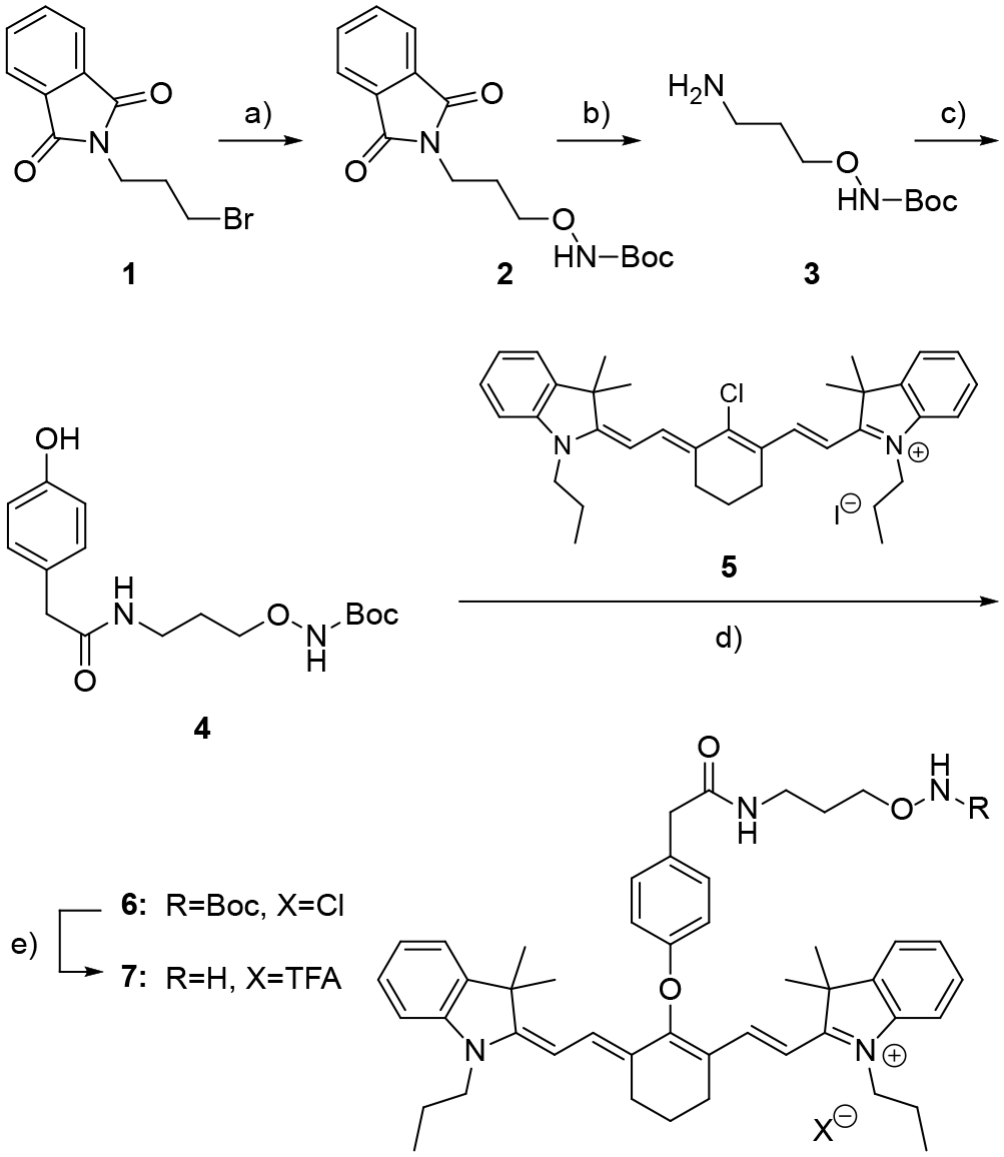
Synthesis of NIR probe 7. Reagents and conditions: a) *N*-Boc hydroxylamine, DBU, DCM, r.t., 68%; b) hydrazine monohydrate, MeOH, r.t., 88%; c) 4-hydroxyphenylacetic acid, EDC·HCl, HOBt·H_2_O, DMF, r.t. 80%; d) 5, NaH, DMF, r.t. 55%; and e) TFA, DCM, r.t. 71%.

The synthetic route used to prepare the fluorescent probe, **7**, is shown in Fig 2. Compound **3** was synthesized using a modified, two-step procedure, which afforded a higher yield than previously reported [35]. Briefly, compound **2** was prepared by a substitution reaction between *N*-(3-bromopropyl)phthalimide (**1**) and *N*-Boc-hydroxylamine in the presence of 1,8-diazabicyclo[5.4.0]undec-7-ene (DBU). The phthalimide protecting group was removed with hydrazine to afford **3**. We observed that the yield of this reaction nearly doubled (49% vs. 88%) when the reaction mixture was diluted from 400 mM to 50 mM, possibly by reducing intermolecular polymerization. An *N*-(3-dimethylaminopropyl)-*N*′-ethylcarbodiimide hydrochloride (EDC) and 1-hydroxybenzotriazole hydrate (HOBt) mediated amide coupling reaction between **3** and hydroxyphenylacetic acid gave **4** in excellent yield. The commercially available cyanine dye, **5**, was then reacted with **4** in the presence of NaH to give the substitution product, **6**. A trifluoroacetic acid (TFA) mediated Boc deprotection of **6** afforded **7** with a 19% overall yield.

#### Fluorescence characterization of 7

**Fig 3:**
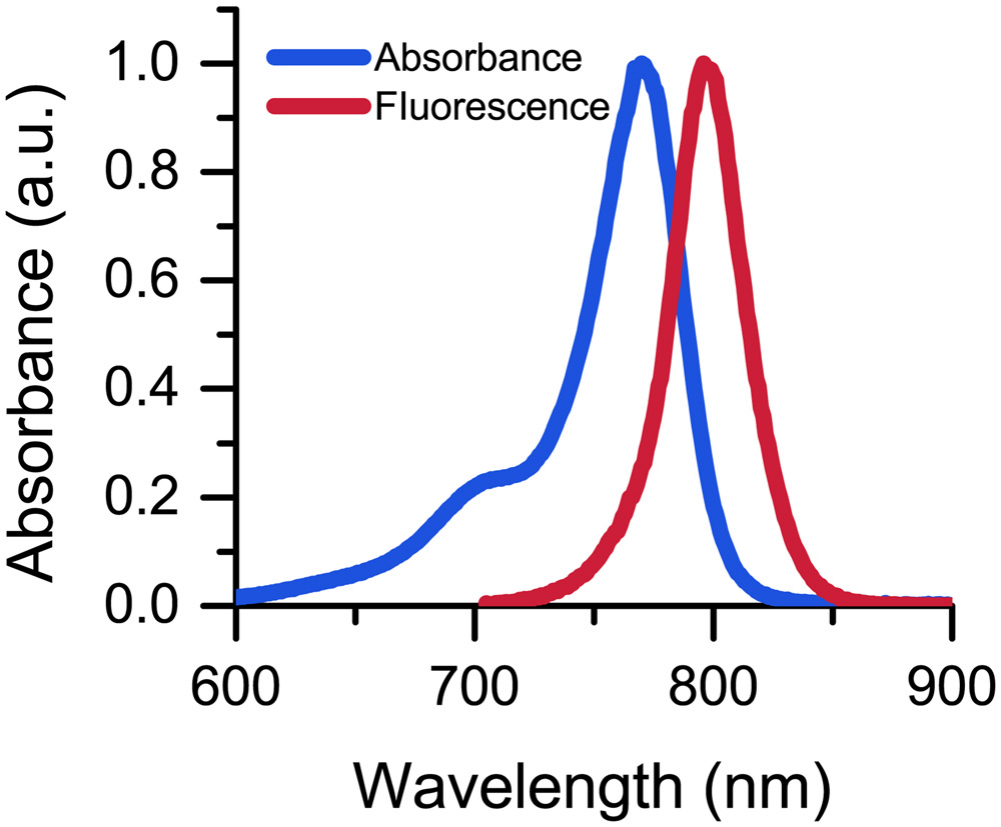
Normalized absorbance and fluorescence emission spectra for 7. Absorbance (blue) and fluorescence (red) measured in EtOH.

The absorption and fluorescence properties of **7** were then evaluated in relatively nonpolar (CHCl_3_), polar aprotic (MeCN), and polar protic (EtOH and H_2_O) solvents. No solvatochromic shifts were observed between the spectra in these solvents (Fig 3) with the absorption maximum near 770 nm and emission maximum near 790 nm. Likewise, the quantum yields of **7** in these solvents compare favorably with other cyanine dyes (Table 1) [36,37].

**Table 1:** Comparison of the optical properties of 7 in various solvents.

Solvent	*ε* (M^−1^cm^−1^)	λ_abs_ (nm)	λ_em_ (nm)	Φ (%)
H_2_O	140,000	767	790	6.6
EtOH	127,000	770	796	39.3
MeCN	154,000	767	789	48.7
CHCl_3_	Not determined	770	791	38.2

### SSB assay to evaluate AP site binding ability

**Fig 4:**
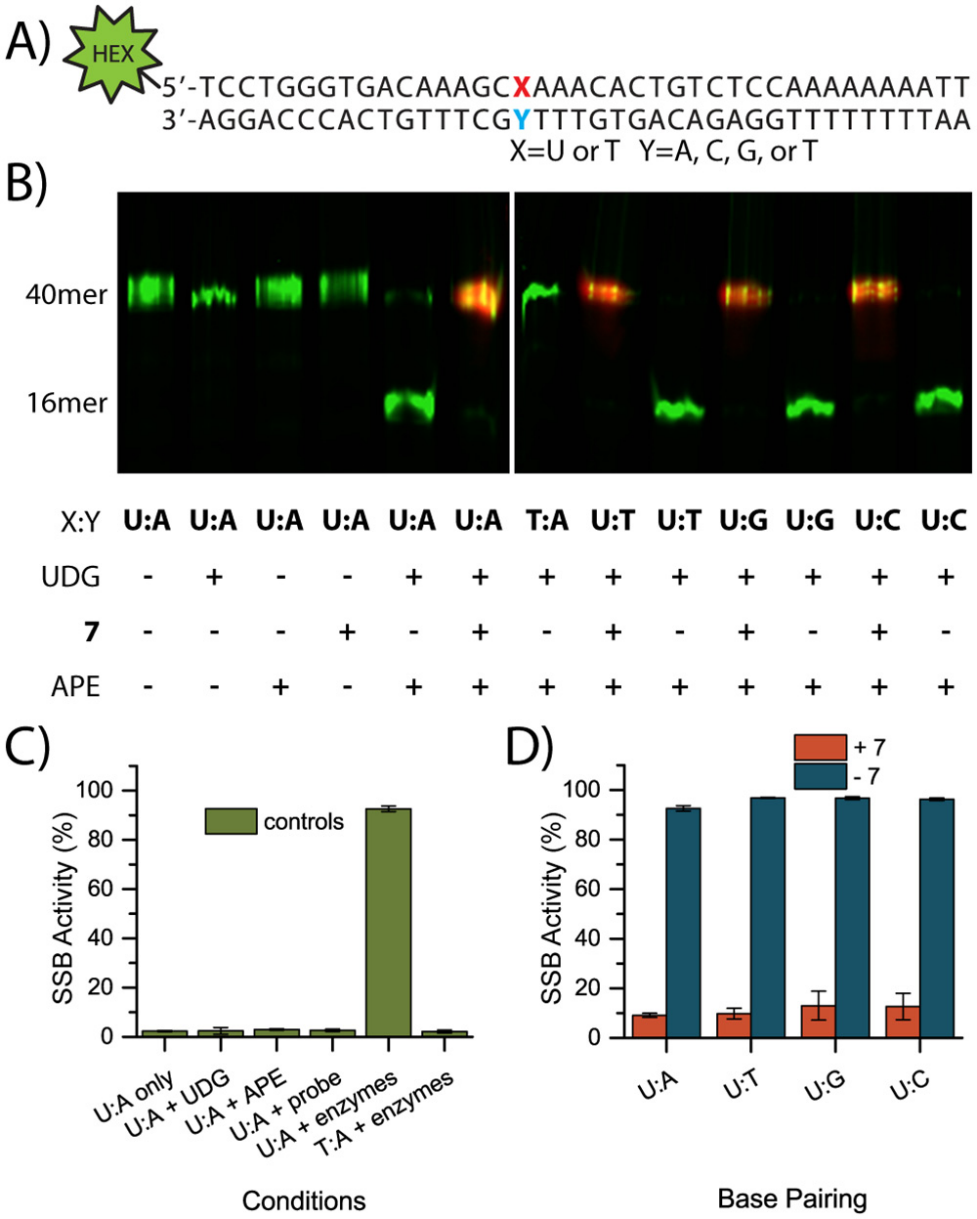
Summary of SSB activity assay. (A) 5’-HEX labeled dsDNA 40mer used for fluorescence-based cutting assay; (B) SSB assay visualized by denaturing gel electrophoresis to separate intact 40mer from 16mer. HEX labeled DNA is shown in green and compound **7** is in red. (C) Analysis of the gel electrophoresis controls with SSB activity % = (Fl 16mer)/(Fl 16mer + Fl 40mer) x 100. (D) Analysis of gel electrophoresis with **7** or vehicle control.

After evaluating the fluorescence properties of **7**, its ability to bind to AP sites was examined. We developed an assay using a fluorescently tagged dsDNA (double stranded DNA) oligonucleotide [38] with a single uracil incorporated into the labeled strand (Fig 4A). A green emitting hexachlorofluorescein (HEX) label was incorporated at the 5’ end of the sense strand in order to avoid overlapping with the NIR fluorecent emission of **7**. We expected that treatment of this oligomer with UDG would generate an AP site and subsequent treatment with APE would produce a single strand break (SSB, Fig 1, path A). This break could be visualized by denaturing gel electrophoresis to reveal a 16mer single strand DNA (ssDNA) instead of the 40mer parent (Fig 4B). However, we hypothesized that if the oligomer were treated with **7** prior to APE treatment, the covalent **7**:AP site lesion would not be recognized by APE as a substrate and no SSB would form (Fig 1, path B).

**Fig 5:**
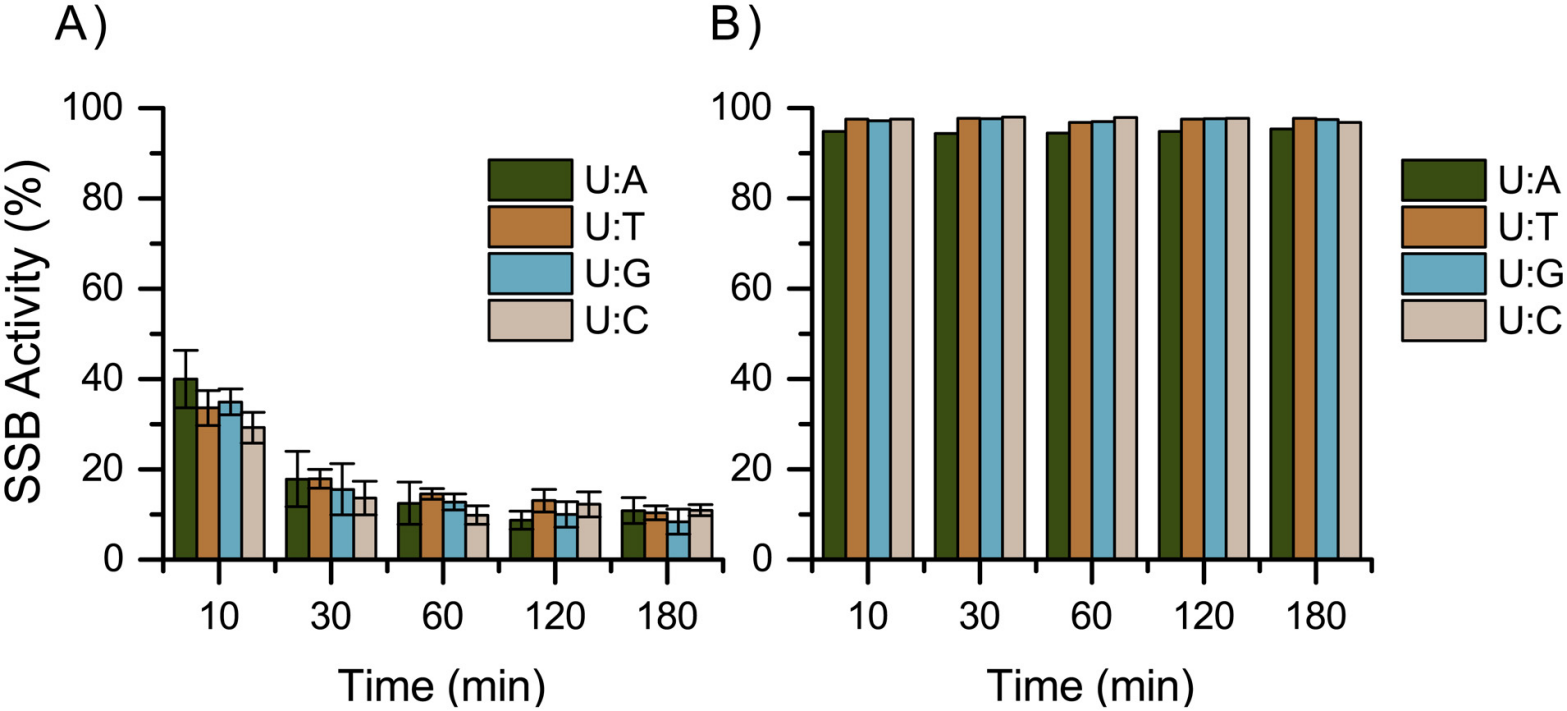
Evaluation of base pairing on SSB activity. Time course represents incubation time of U:X DNA before APE addition with UDG and (A) compound **7** or (B) with vehicle control.

To validate the assay, we first ran a series of controls. As the U:A base pair is most likely to occur when dUTP is incorporated in place of dTTP [10], we used this as our primary substrate. As expected, we observed no SSB activity when UDG and/or APE were omitted or when a T:A substrate oligomer was used. Likewise, U:A-paired DNA treated with **7** alone (no enzymes) did not lead to a SSB, indicating that the probe itself has no SSB activity. Instead, when the oligomer was treated with both UDG and APE in the absence of **7**, we saw nearly quantitative breaking (Fig 4B and 4C). When U:A-paired DNA was treated with **7** together with UDG and APE, the SSB activity was reduced. When the NIR channel emission was overlaid with the green channel (HEX) emission, colocalization is observed only in 40mer strands treated with **7** and both enzymes but not in DNA treated with **7** alone, indicating that **7** is binding to the AP site and not elsewhere along the DNA (Fig 4B). The base opposite to the uracil does not appear to affect the binding of **7** to the AP site (Fig 4D and Fig 5).

**Fig 6:**
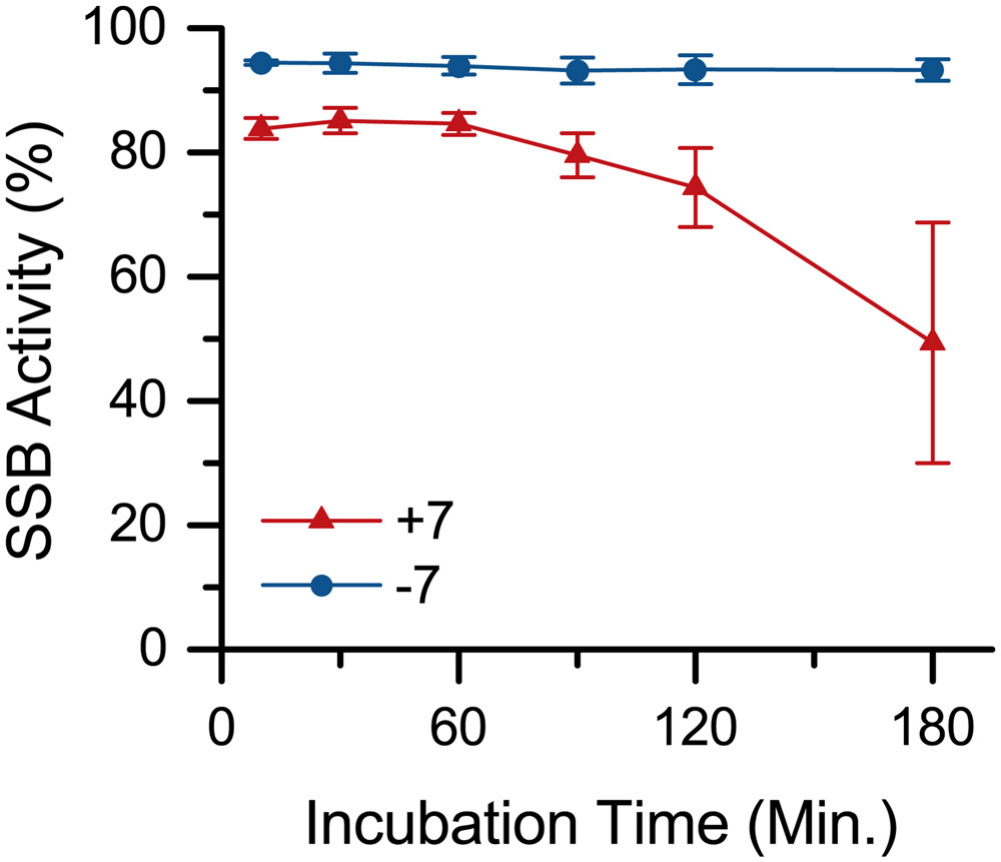
Evaluation of APE inhibition by 7. APE shows mild inhibition by **7** when incubated together before addition of AP-DNA. The SSB activity assay used 60 min APE incubation after treatment of AP-DNA with **7**, which here shows no detectable inhibition. APE is inhibited about 50% after 3 h incubation. The baseline reduction in cutting is due to competition of APE and **7** for the AP-DNA substrate.

We determined if **7** could inhibit the activity of the enzymes and thereby complicate the results on the assay. While the order of reagent addition in the assay may be structured to avoid enzyme inhibition, compound **7** is envisioned as an imaging agent in living cells where the enzymes are constantly present. To test the possible inhibition of **7** towards APE, the probe and enzyme were incubated together for a variable period of time then incubated for a fixed period of time with AP DNA (U:A DNA pretreated with UDG). If the two species were only competing for substrate, then the extent of cutting observed would be independent of the APE+**7** incubation time. Conversely, if **7** inhibits the action of APE, then the extent of cutting would vary as a function of APE+**7** incubation time. In either case, some baseline reduction in cutting was expected as **7** and APE competed for substrate. As shown in Fig 6, there was a change in cutting as APE and **7** incubation time increased, indicating that **7** did have inhibitory effects on APE. However, this effect was only observed after 60 minuntes. Thus, the one hour incubation of APE in the cutting assay is not expected to be negatively impacted by the presence of **7**. An additional APE inhibition study using a tetrahydrofuran (THF) base in place of the AP site were conducted and suggest moderate inhibition of APE by **7** for this substrate. These data are described in S3 Fig and S1 File.

**Fig 7:**
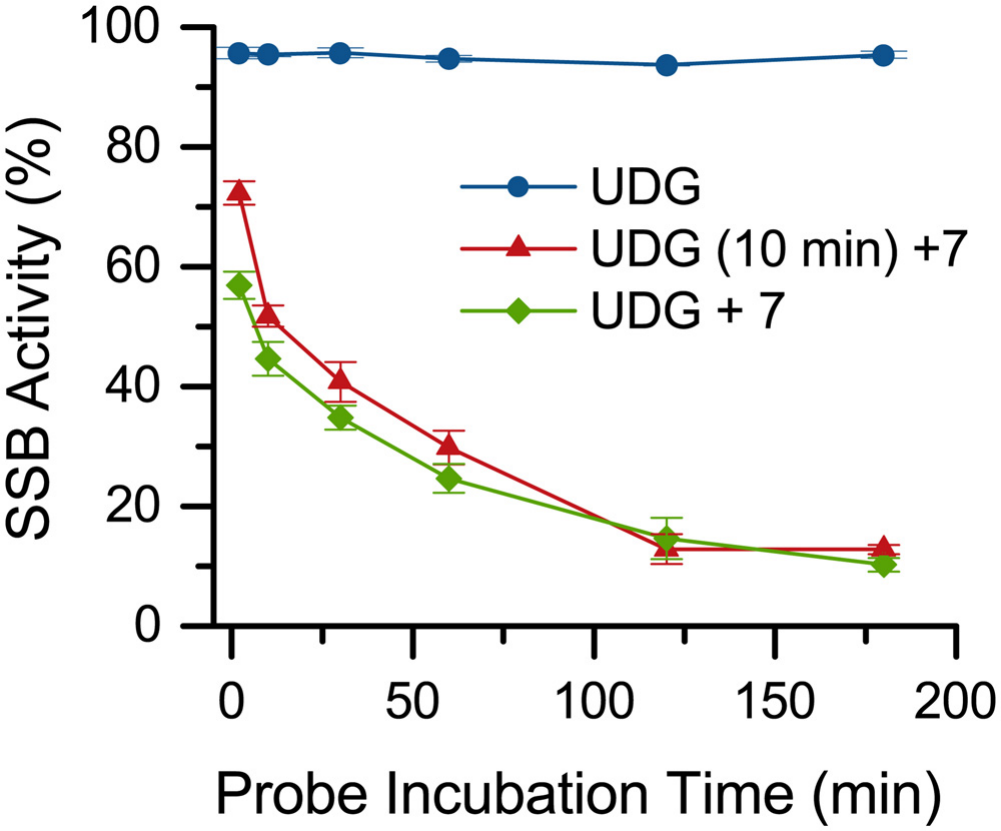
Evaluation of UDG inhibition by 7. Time course represents incubation time of U:A DNA before APE addition with UDG alone, 10 minutes UDG pretreatment then **7**, and UDG and **7** without pretreatment.

Likewise, the potential of **7** to inhibit UDG, either through direct interaction with the enzyme or by binding to DNA and blocking the uracil substrate, was tested. To accomplish this, U:A DNA was incubated with UDG for a fixed time prior to addition of **7** (Fig 7, UDG (10min)+**7**). In this case, **7** could not interfere with the activity of UDG. These data were compared to samples where U:A DNA was treated simultaneously with **7** and UDG (Fig 7, UDG+**7**). No significant difference in cutting activity was observed between these conditions indicating that **7** does not inhibit UDG activity.

#### Comparison of 7 to MX and ARP in SSB assay

**Fig 8:**
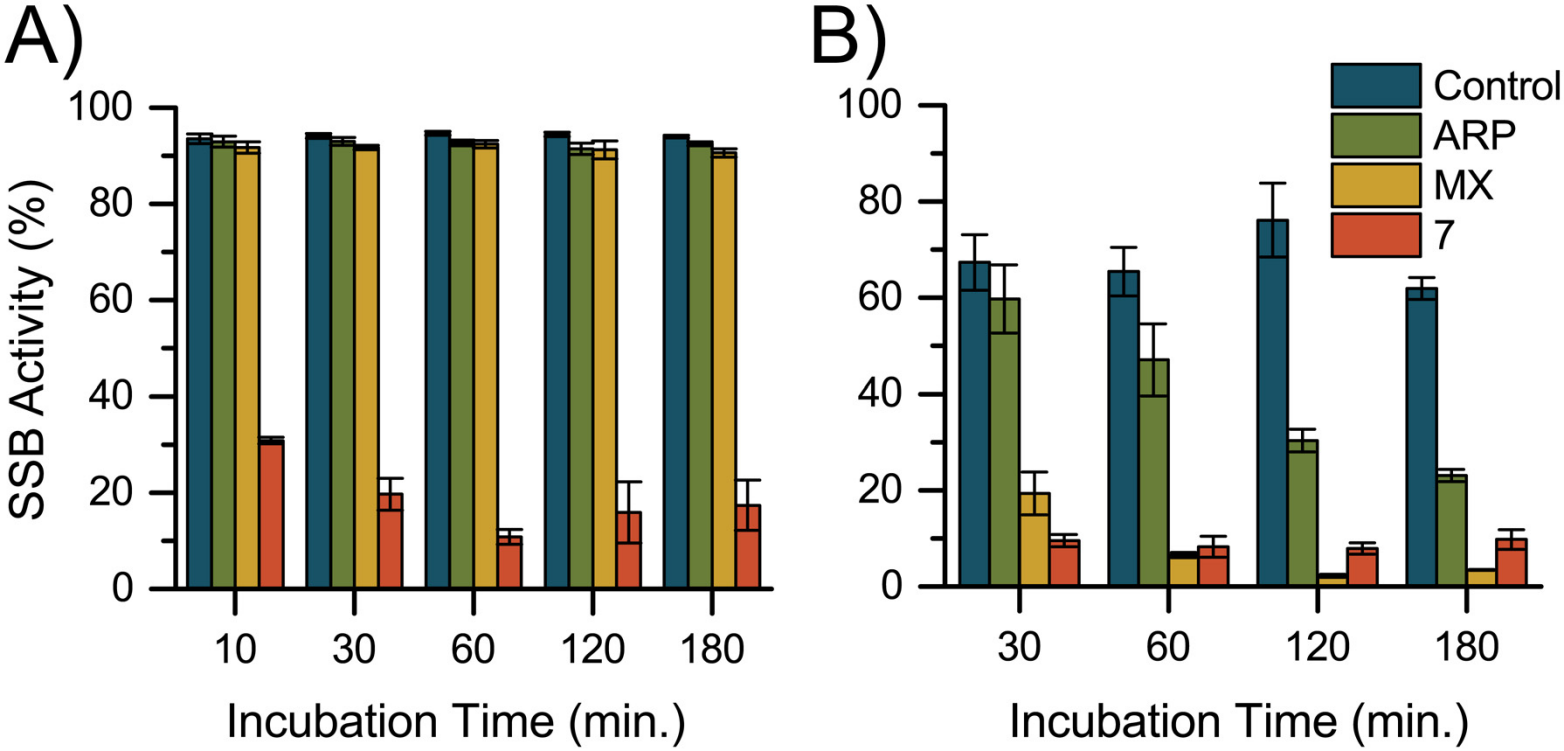
SSB activity assay comparison of ARP, MX, and 7. (A) ARP (1 nmol), MX (1 nmol), and **7** (1 nmol) with UDG (5 units) and U:A DNA (5pmol) as a function of incubation time prior to APE (10 units) addition. (B) ARP (200 nmol), MX (200 nmol), and **7** (2 nmol) with UDG (5 units) and U:A DNA (5 pmol) as a function of incubation time prior to APE (1 unit) addition.

We then compared **7** to two AP site-binding probes that have previously been developed, ARP and MX [30,31,32]. Under the same assay conditions of the control experiments (1 nmol probe, 5 pmol DNA, 1X APE), we observed that **7** rapidly blocked the SSB activity of APE while ARP and MX failed to block almost any relative to the control (Fig 8A). To ensure that this dramatic difference was not an artifact of the assay, we determined conditions under which ARP and MX could be observed to block APE activity. Therefore, we increased the amounts of ARP and MX (100x relative to **7**) and decreased the amount of APE (0.1X), as the enzyme and probes compete for the AP site substrate. Under these conditions, we observed that ARP and MX were able to block the SSB activity of APE (Fig 8B). Thus, we concluded that **7** binds to AP sites more potently than ARP and MX do in the reaction conditions employed here.

### Characterization of AP site binding in calf thymus DNA

**Fig 9:**
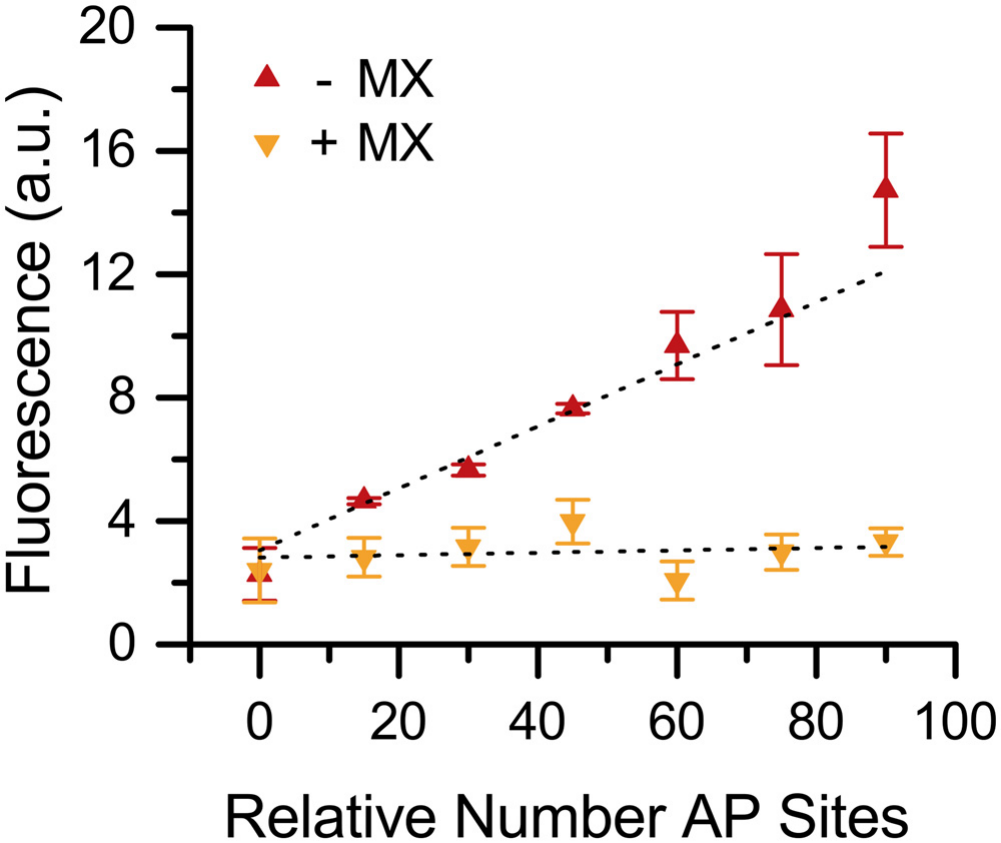
Fluorescence response and MX blocking of 7. Calf thymus DNA treated with heat and acid for increments of 15 min to give a linear relative number of AP sites. AP DNA was then pretreated with MX (50 mM, +MX) or NaCl (50 mM, -MX) followed by incubation with **7** (0.05 mM). Fluorescence measures show a linear response to **7** when not blocked by MX.

Having established the ability of **7** to recognize and bind AP sites, we also demonstrated that the fluorescence is proportional to the quantity of lesions present in DNA. Following a procedure described by Lindahl and Nyberg [39], calf thymus DNA was heated at 70°C in citrate buffer (pH 5.0). This treatment generates a number of AP sites that increases linearly with time. This DNA was incubated with **7** and subsequently purified by ethanol precipitation to remove unreacted **7**. A linear relationship was observed between the fluorescence intensity of **7** and DNA heat/acid treatment time, reflecting the increase of AP sites and indicating that **7** can report on the quantity of AP sites in a genomic sample (Fig 9). When DNA was pretreated with a large excess of MX (1000x), the fluorescent intensity was significantly reduced, suggesting that MX was able to block the binding of **7.** These studies indicated that **7** binds selectively to AP sites in DNA.

**Fig 10:**
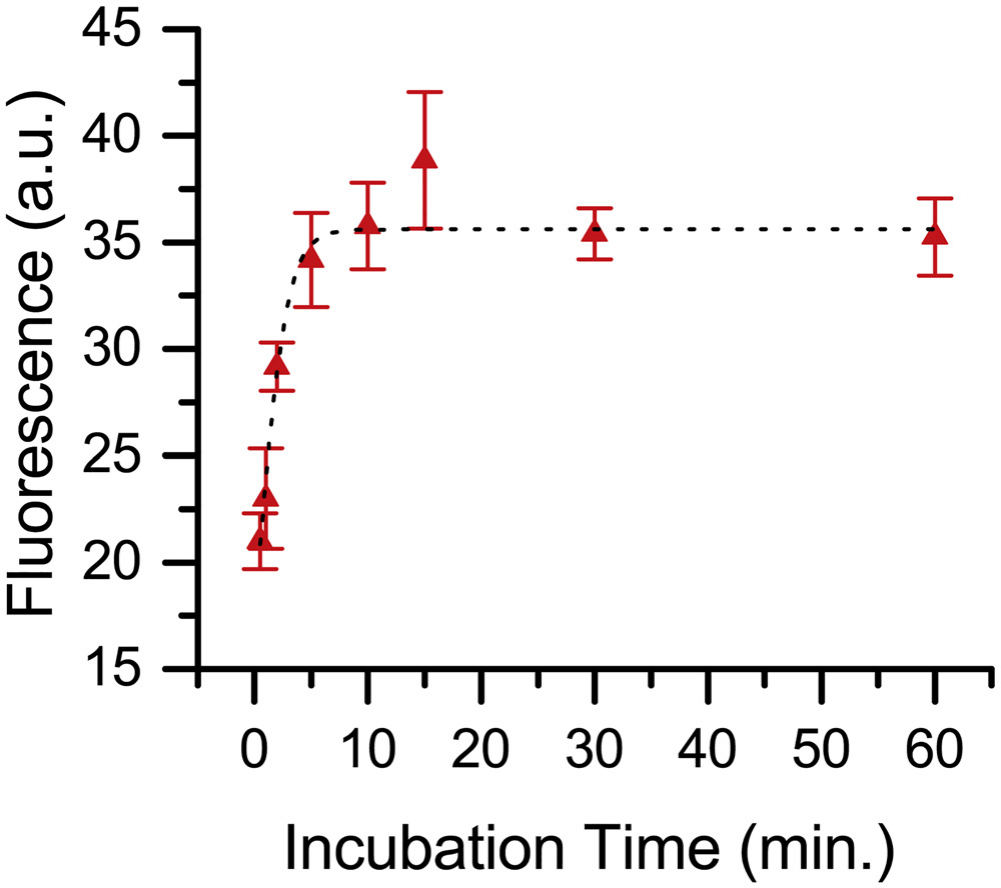
Time course of 7 binding to calf thymus AP DNA.

**Fig 11:**
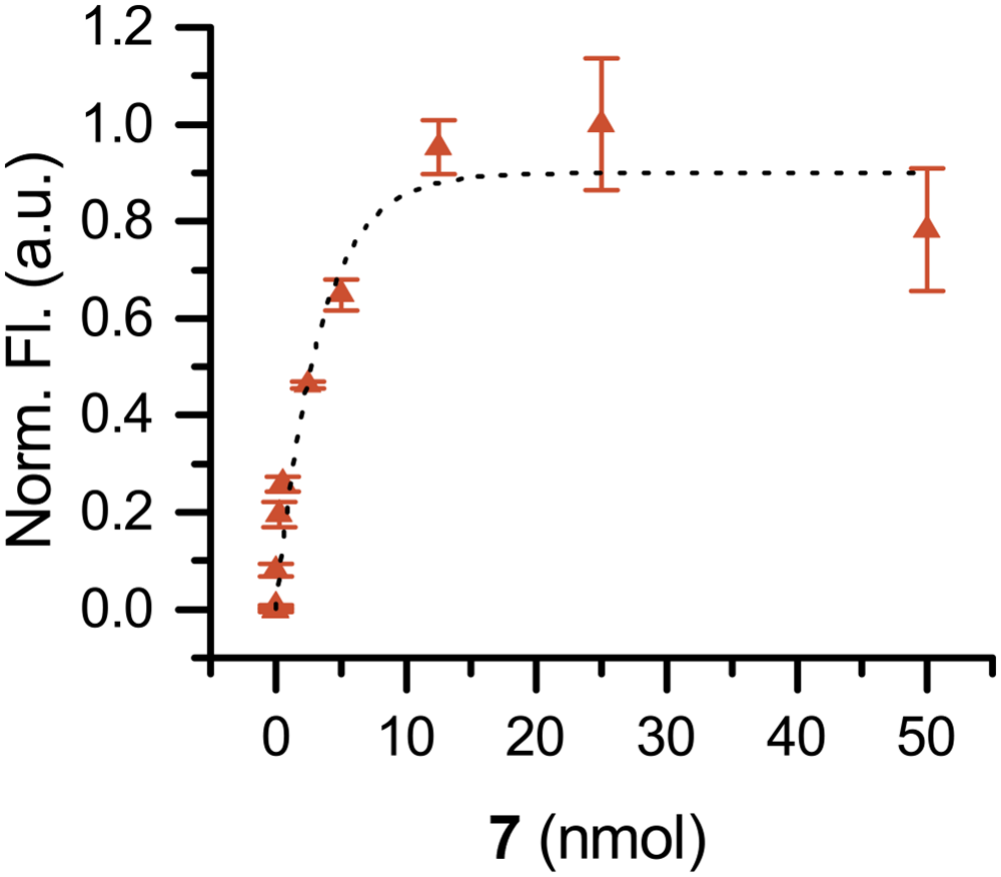
Dose-response of 7 in calf thymus AP DNA.

The time course of heat/acid treated DNA was measured to determine directly the rate of **7** binding to AP sites. AP DNA and **7** were incubated at 37°C for a variable time then quickly precipitated with purified cold ethanol. The fluorescence was measured and from these data (Fig 10), we calculated reaction half-life (t_1/2_ = 2.6 min) and observed the reaction was complete after approximately 10 min. In addition, a dose-response curve was generated by varying the quantity of **7** and maintaining a constant quantity of AP DNA. The fluorescence was measured and from these data (Fig 11), we calculated ED_50_ = 2.3 nmol.

#### Comparison of 7 to MX and ARP calf thymus binding

**Fig 12:**
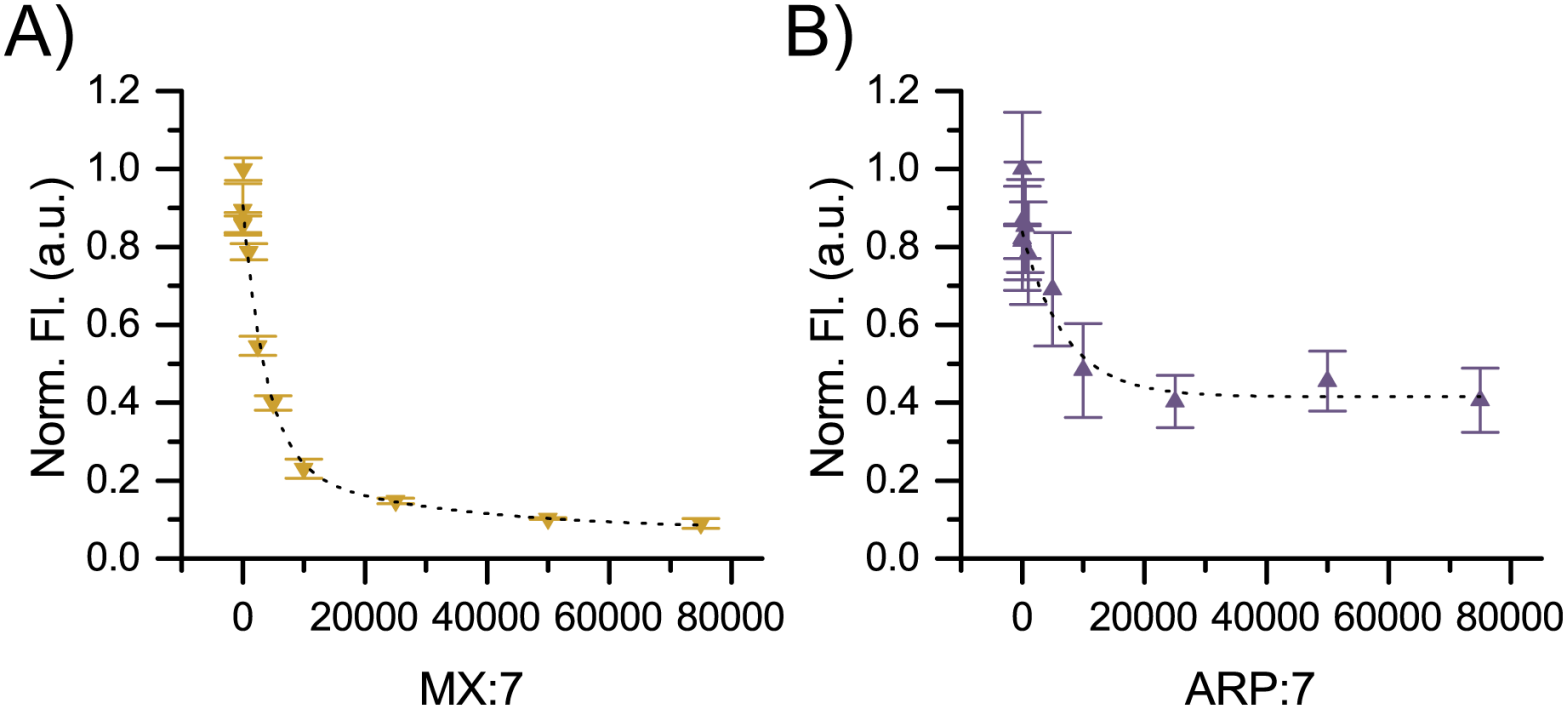
Competition studies between 7 and MX or ARP in genomic DNA. (A) MX or (B) ARP for AP sites in calf thymus DNA treated with heat and acid for 45 min are shown. The molar ratio of probe to **7** was increased by increasing [probe] and maintaining a constant [7]. ED_50_ values were calculated from the fittings to be 2600-fold excess for ARP and 3000-fold excess for MX.

To verify the results of the SSB assay (Fig 8), we performed a competition assay between **7** and either ARP or MX. Keeping the molar concentration of **7** constant, the molar concentration of ARP/MX was adjusted so that the MX or ARP:**7** ratio ranged from 0.01 to 75,000. These solutions of MX or ARP/**7** were added to the DNA for 1 h at 37°C. Fluorescence was measured following ethanol precipitation to remove unbound probes. These results (Fig 12) indicate that the ED_50_ of **7** was at 3000x excess of MX and 2600x excess of ARP. We presume the increased hydrophobicity of **7** over MX and ARP as well as a mild Coulombic interaction may favor an association of **7** with DNA over bulk solution and contribute to this drastic difference.

### Detection of AP sites in colon cancer cells

**Fig 13:**
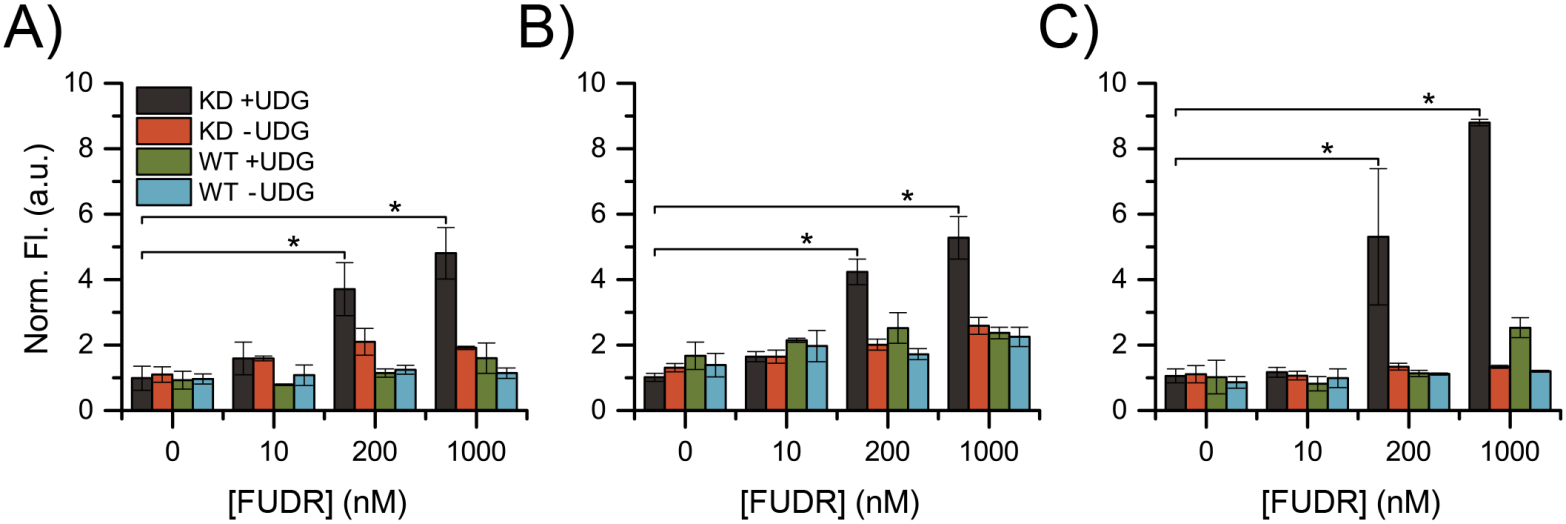
Detection of AP sites in DNA isolated from FUDR treated DLD1 cells. DNA extracted from UDG knockdown (KD) and control (WT) cells after (A) 24 h, (B) 48 h, and (C) 72 h of continuous FUDR exposure. Purified DNA was treated in vitro with UDG (+ UDG) or vehicle control (-UDG). * Indicates significance (p<0.001) using a one-way ANOVA test.

Finally, we sought to demonstrate that **7** can report on physiologically relevant quantities of AP sites. To this end, we prepared a shRNA UDG knockdown in the DLD1 colon cancer cell line. DLD1 cells (ATCC #CCL-221) were obtained from the laboratory of Sanford Markowitz at Case Western Reserve University. This knockdown cell line (KD) and its matched, shRNA scrambled control line (WT) were treated with the antimetabolite, 5-fluoro-2′-deoxyuridine (FUDR or floxuridine), for 24, 48, and 72 hours. An antimetabolite like aminopterin, FUDR is used clinically to treat colon cancer [40]. FUDR induces DNA damage in two ways: first, by inhibiting thymidylate synthase [41,42] and decreasing the thymine pool leading to uracil misincorporation; and second, by cellular activation then direct incorporation of 5-fluorodeoxyuracil (FUra) into the DNA chain [43,44]. Both uracil and FUra lesions are repaired by UDG [43,45,46,47]. Therefore, we hypothesized that following FUDR treatment the KD would accumulate lesions while the WT would repair them effectively. Isolation of DNA from a cell lysate followed by in vitro treatment with purified UDG enzyme (or vehicle control) and **7** would allow us to visualize DNA damage and repair in the DLD1 cell line. Fluorescence measurements of **7** revealed that the KD cells did accumulate damage that was repaired in vitro while WT cell lines did not (Fig 13). After 72 h of continuous FUDR exposure, the UDG treated KD showed nearly a 9-fold increase in fluorescence intensity, indicating that **7** can report on physiologically relevant concentrations of AP sites. Additionally, this experiment demonstrates a tool that can be used to quantify uracil lesions in DNA. This could be useful to understand the mechanism and response of drug treatment in cancer cells.

Aliquots of each sample were set aside following exogenous UDG treatment but before addition of **7**. These samples were analyzed by the ARP-based DNA Damage Quantification Kit by Dojindo (S1 Fig). The results of this assay showed a larger background and lower sensitivity than compound **7**, but confirmed the trends observed in Fig 12. One advantage of **7** over ARP is that **7** contains both the AP site-binding moiety and the detection tool. ARP binds to AP sites but relies on two additional reagents, HRP-streptavidin and a substrate solution, for detection. Further, in the ARP assay, ten steps that require transfer or dilution of the DNA, which introduce error in the final DNA concentration, follow DNA concentration adjustment to 100 μg/mL (S2 Fig). In the assay with **7**, DNA concentration can be adjusted just before analysis to minimize error. The ARP assay takes at least two days to complete but must be analyzed within one hour of addition of the final substrate solution. Conversely, the assay with **7** takes approximately 3 hours and samples are stable for at least two days when stored in the dark (we did not examine stability longer than two days). Therefore, we believe that compound **7** is an attractive alternative to ARP for AP site quantification.

FUDR and DLD1 KD cells were able to elucidate the accumulated effect of a DNA damaging drug over time. However, UDG KD cell lines are not expected in nature. Measurement of the accumulated effect of a drug may not be possible for all purposes. Thus, the ability of **7** to measure AP sites at a single time point was explored.

Methyl methanesulfonate (MMS) is a methylating agent used to induce AP sites in cells. An MMS dose-response in DLD1 WT cells was performed for a 3 h treatment time. The WT cell line has normal BER and is expected to actively undergo repair in response to the drug. Following drug treatment, DNA was extracted by phenol-chloroform and incubated with **7** for 1 h. Fluorescence was measured following ethanol precipitation purification.

**Fig 14:**
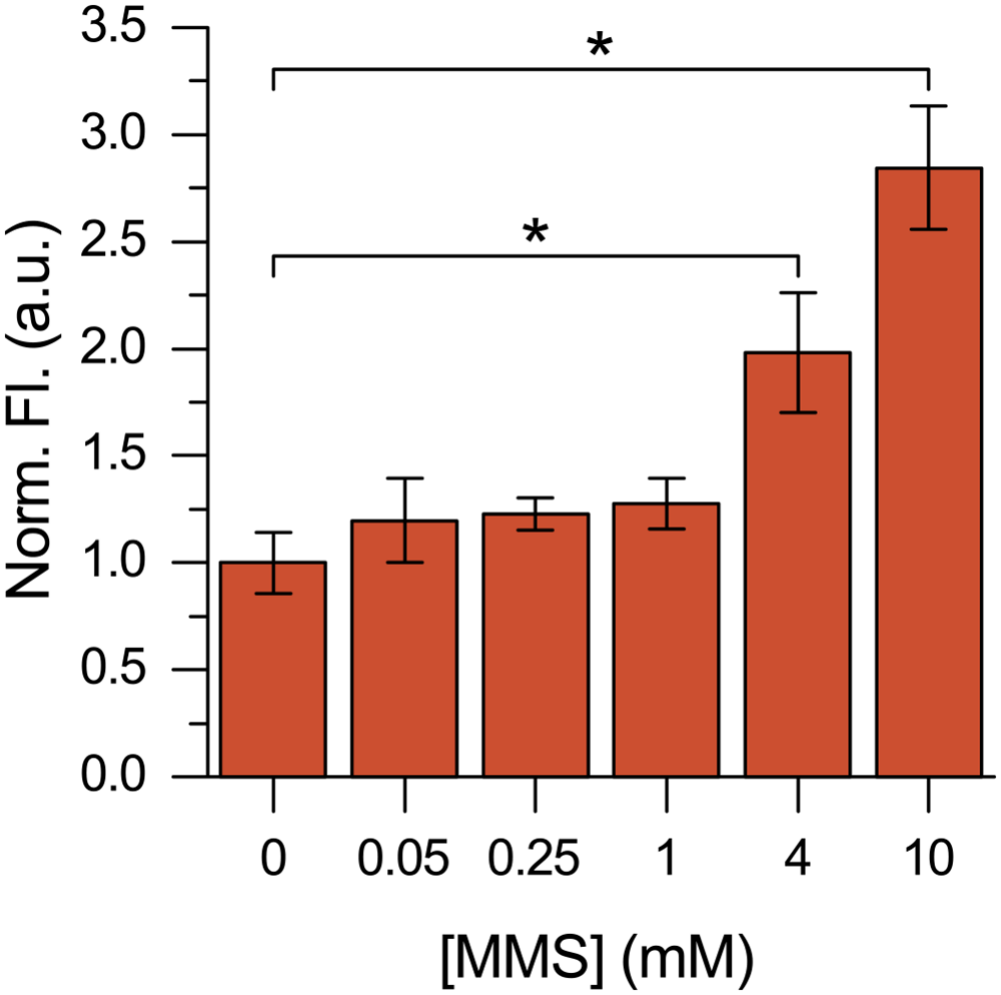
Detection of AP sites in DNA isolated from MMS treated DLD1 cells. DNA extracted from DLD1 WT cells after 3 h of continuous MMS exposure. Purified DNA was treated with **7** without enzyme pretreatment. * Indicates significance (p<0.001) using a one-way ANOVA test.

The data indicate that **7** was sensitive to the quantity of AP sites induced by mM concentrations of MMS (Fig 14). These results suggest that **7** can detect physiological concentrations of AP sites at a single time point.

## Conclusions

In conclusion, we have developed a fluorescent probe for evaluating DNA damage and repair. We have demonstrated the use of this probe to quantify AP site and uracil lesions in DNA. Based on our findings, our probe binds to AP sites with higher potency than MX and ARP. A side-by-side comparison of our assay and the commercialized ARP assay (see S2 Fig) shows our method as a simplified and straightforward alternative to ARP with less potential to introduce error. A distinct advantage over ARP in that **7** has the potential to be used directly to detect DNA damage in vivo. While near infrared fluorescence is a prerequisite to in vivo imaging, additional pharmacokinetic properties such as metabolic activity, tumor uptake, retention, and clearance, also need to be considered as various attributes of in vivo studies. The studies of these properties are on the way and will be reported separately.

## Materials and Methods

### Chemistry

#### Synthesis

Compound **7** was synthesized according to Fig 2 tert-Butyl 3-(1,3-dioxoisoindolin-2-yl)propoxycarbamate (**2**) and tert-butyl 3-aminopropoxycarbamate (**3**) were prepared according to a literature procedure [35].

tert-butyl 3-(2-(4-hydroxyphenyl)acetamido)propoxycarbamate (**4**). 4-hydroxyphenylacetic acid (350 mg, 2.30 mmol, 1.05 eq), **3** (416 mg, 2.19 mmol, 1.00 eq), EDC·HCl (629 mg, 3.28 mmol, 1.50 eq), and HOBt·H_2_O (503 mg, 3.28 mmol, 1.50 eq) were added to an oven-dried 50 mL round bottom flask fitted with a magnetic stir bar. The solids were dissolved in dry dimethylformamide (DMF, 20.0 mL) and the reaction was stirred at room temperature under argon for 24 h. The reaction was then diluted with water (100 mL) and ethyl acetate (EtOAc, 50 mL) and extracted. The aqueous layer was extracted twice more with EtOAc (25 mL). The combined organic layers were washed with water (2 x 40 mL) and brine (40 mL) then dried over MgSO_4_, filtered, and concentrated. The crude residue was diluted in a trace amount of dichloromethane (DCM) and purified by silica gel chromatography with a mobile phase of pure DCM then gradually increasing polarity to 3:2 DCM/acetonitrile (MeCN). This product was then further purified by diluting it in EtOAc (25 mL) and washing with sat. NaHCO_3_ (3 x 25 mL) and brine (1 x 25 mL). The organic layer was dried over MgSO_4_, filtered, and concentrated to afford pure **4** as a white solid (527 mg, 74%). R_f_ = 0.23 (DCM/MeCN, 3:2); ^1^H NMR (400 MHz, CDCl_3_): *δ* = 8.64 (br s, 1H), 8.21 (br s, 1H), 7.34 (br s, 1H), 7.07 (d, J = 8.6 Hz, 2H), 6.74 (d, J = 8.6 Hz; 2H), 3.84 (t, J = 5.6 Hz, 2H), 3.46 (s, 2H), 3.35 (dt, J = 6.0, 12 Hz, 2H), 1.71 (tt, J = 6.0, 5.6 Hz, 2H), 1.47 (s, 9H); ^13^C NMR (100 MHz, CDCl_3_): *δ* = 172.8, 157.3, 155.7, 130.0, 125.7, 115.6, 81.6, 74.6, 42.4, 37.0, 28.0, 26.8;

2-((E)-2-((E)-3-((E)-2-(3,3-dimethyl-1-propylindolin-2-ylidene)ethylidene)-2-(4-(2,2-dimethyl-4,11-dioxo-3,6-dioxa-5,10-diazadodecan-12-yl)phenoxy)cyclohex-1-en-1-yl)vinyl)-3,3-dimethyl-1-propyl-3H-indol-1-ium chloride (**6**). NaH (14.1 mg, 0.588 mmol, 1.70 eq) and **4** (129 mg, 0.398 mmol, 1.15 eq) were added to an oven-dried 25 mL round bottom flask fitted with a magnetic stir bar. Dry DMF (3.0 mL) was added and the mixture was stirred under argon at room temperature for 30 min. Meanwhile, IR 780 iodide (**5**, 230.1 mg, 0.345 mmol, 1.00 eq) was added to a 15 mL oven-dried heart-shaped flask fitted with a magnetic stir bar. DMF (5.0 mL) was added and **5** was stirred under argon at room temperature shielded from light. After 30 min, the solution of **5** was transferred to the NaH mixture via syringe. The heart-shaped flask was rinsed with dry DMF (4 x 2 mL) and the rinsate was added to the reaction mixture via syringe. The reaction was stirred at room temperature under argon in the dark for 5 h. The reaction was quenched with water (100 mL) and 10% NH_4_Cl (aqueous, 50 mL). The aqueous mixture was extracted with DCM (1 x 100 mL, 1 x 25 mL) until the aqueous layer remained colorless. The combined organic layers were washed with water (2 x 50 mL) and brine (1 x 50 mL), dried over MgSO_4_, filtered, and concentrated. The crude residue was diluted in a minimal amount of eluent and purified by silica gel chromatography with a mobile phase of 1:4:35 methanol (MeOH)/MeCN/DCM gradually increasing to 2:3:15 MeOH/MeCN/DCM. Impure fractions were concentrated and this chromatographic method was repeated one time. Pure fraction were combined and concentrated to afford **6** as an emerald solid (163 mg, 55%).R_f_ = 0.15 (MeOH/MeCN/DCM, 1:3:16); ^1^H NMR (400 MHz, CDCl_3_): δ = 8.94 (br s, 1H), 8.78 (br t, J = 5.6 Hz, 1H), 7.94 (d, J = 14.0 Hz, 2H), 7.55 (d, J = 8.4 Hz, 2H), 7.35–7.28 (m, 4H), 7.19 (dd, J = 7.6, 7.2 Hz, 2H), 7.05 (d, J = 8.0 Hz; 2H), 6.95 (d, J = 8.8 Hz, 2H), 5.95 (d, J = 14.0 Hz, 2H), 3.96 (t, J = 7.2 Hz, 4H), 3.92 (t, J = 5.6 Hz, 2H), 3.62 (s, 2H), 3.28 (dt, J = 6.0, 6.0 Hz, 2H), 2.67 (dd, J = 6.0, 5.6 Hz, 4H), 2.04 (dd, J = 6.0, 5.6 Hz, 2H), 1.86 (tq, J = 7.6, 7.2Hz, 4H), 1.70 (tt, J = 6.0, 5.6Hz, 2H), 1.45 (s, 9H), 1.32 (s, 12H), 1.04 (t, J = 7.6 Hz, 6H); ^13^C NMR (75 MHz, CDCl_3_): δ = 172.1, 171.6, 164.8, 158.3, 156.8, 142.3, 141.9, 140.8, 131.3, 131.2, 128.3, 124.9, 122.1, 121.7, 114.1, 110.2, 99.2, 80.3, 73.5, 48.9, 45.6, 42.3, 35.7, 28.1, 27.6, 27.2, 24.1, 20.9, 20.5, 11.4; MS-ESI: m/z [M]^+^ calculated for C_52_H_67_N_4_O_5_^+^: 827.51, found: 827.47.

2-((E)-2-((E)-2-(4-(2-((3-(aminooxy)propyl)amino)-2-oxoethyl)phenoxy)-3-((E)-2-(3,3-dimethyl-1-propylindolin-2-ylidene)ethylidene)cyclohex-1-en-1-yl)vinyl)-3,3-dimethyl-1-propyl-3H-indol-1-ium 2,2,2-trifluoroacetate (**7**). **6** (23.0 mg, 0.027 mmol, 1.00 eq) was dissolved in DCM (1.0 mL). TFA (1.0 mL, 13.0 mmol, 1.48 g/mL, 480 eq) was added and the solution immediately turned from dark green to dark red. The reaction was stirred for 1 h in the dark. The solvents were removed by rotary evaporation to afford pure **7** as an emerald green solid (16 mg, 71%). R_f_ = 0.21 (MeOH/DCM, 1:9); ^1^H NMR (600 MHz, CDCl_3_): 8.93–8.20 (br s, 2H), 8.0–7.87 (m, 3H), 7.37–7.26 (m, 6H), 7.19 (dd, J = 7.8, 7.2 Hz, 2H), 7.02 (d, J = 7.8 Hz, 2H), 6.97(J = 8.4 Hz, 2H), 5.93 (bs d, J = 12.6 Hz, 2H), 4.09 (br s, 2H), 3.91 (bs t, J = 7.2 Hz, 4H), 3.48 (br s, 2H), 3.23 (br m, 2H), 2.65 (br s, 4H), 2.02 (br m, 2H), 1.83 (tq, J = 7.2, 7.2, 4H), 1.67 (br s, 2H), 1.30 (s, 12H), 1.02 (t, J = 7.2 Hz, 6H); ^13^C NMR (150 MHz, CD_2_Cl_2_, -10°C): δ = 174.2, 172.4, 164.7, 161.0, 159.2, 142.5, 141.3, 131.2, 129.6, 128.7, 125.3, 122.6, 122.1, 115.1, 110.7, 99.8, 72.2, 49.3, 46.0, 41.9, 35.8, 27.7, 27.6, 24.4, 21.3, 20.9, 11.7; UV/Vis (EtOH): λ_max_ (ε) = 705 nm (29200 L·mol^−1^·cm^−1^), 770 nm (127000 L·mol^−1^·cm^−1^); HRMS-ESI: m/z [M]^+^ calculated for C_47_H_59_N_4_O_3_^+^: 727.4587, found: 727.4615.

#### Fluorescence quantum yield measurements

Fluorescent quantum yields determined by a comparative method to an indocyanine green (ICG) standard (Φ = 0.132 in EtOH) [36] with refractive index correction according to the equation:

$$\Phi =\Phi_{R}\left(\frac{m}{m_{R}}\right)\left(\frac{n^{2}}{n_{R}{}^{2}}\right)$$

where Φ is the quantum yield; m is the slope of the integrated fluorescence intensity as a function of absorbance maxima; n is the refractive index of the solvent (EtOH, 1.3611; MeCN, 1.3442; H_2_O, 1.333; and CHCl_3_, 1.4459) [48]; and R is the reference sample, ICG in EtOH. Data were collected in a quartz cuvette with a 1 cm path length. Absorbance maxima for each sample were kept at or below 0.2 absorbance units to avoid inner filter effects. Fluorophores were excited at 700 nm and emission spectra integrated from 705 to 1000 nm. Solvents used were spectroscopic grade ethanol and HPLC grade acetonitrile, HPLC grade chloroform, and HPLC grade water. For the water solution, **7** was first dissolved in MeCN and diluted at least 200 fold.

### SSB activity assay

#### General procedures

SSB activity assays were performed on a 40-mer duplex DNA synthesized by IDT with the sequence:

5’-[HEX] TCCTGGGTGACAAAGCXAAACACTGTCTCCAAAAAAAATT

3’-AGGACCCACTGTTTCGYTTTGTGACAGAGGTTTTTTTTAA

where X = uracil or thymine and Y = adenine, cytosine, guanine, or thymine. DNA was diluted in dH_2_O to 500 nM and 5 pmol (10 μL) aliquots were used in each sample. APE (10,000 Units/mL) and UDG (5,000 Units/mL) enzymes and corresponding buffers were purchased from New England BioLabs. UDG storage buffer (10 mM Tris-HCl, 50 mM KCl, 1 mM DTT, 0.1 mM EDTA, 0.1 mg/ml BSA, 50% Glycerol, pH 7.4) and APE storage buffer (10 mM Tris-HCl, 50 mM NaCl, 1 mM DTT, 0.05 mM EDTA, 200 μg/ml BSA, 50% Glycerol, pH 8.0) were prepared according to formulation provided by New England BioLabs to use as blanks, where necessary. The enzymes were not heat inactivated as this was observed to give rise to artifacts. Reaction products were resolved on denaturing 20% polyacrylamide gels (5.3 g urea, 5.0 mL 40% acrylamide, 2.3 mL 5X TBE buffer, 200 μL 10% APS, and 20 μL TEMED). 5X TBE buffer was prepared with tris base (54 g), boric acid (27.5 g), and EDTA (4.65 g) diluted to 1 L in water. Loading dye was prepared (300 μL 10M NaOH, 20 mg bromophenol blue, 9.7 mL formamide) and was added to samples (~ 5 μL) to aid loading and visualization of gel progression. Gel were developed at 300 V for 45 min in the dark and imaged based on the DNA tag on Typhoon Trio + Variable Mode Imager (Amersham Biosciences) in fluorescent mode with 532 nm excitation and 555 nm emission with a 20 nm band pass, PMT set to 400, and pixel size resolution of 100 μm. Gel data were analyzed using ImageQuant software (Amersham Biosciences). DNA cutting was defined as the fluorescence intensity of the 16-mer strand divided by the sum of the fluorescence intensities of the 16- and 40-mer strands. The fluorescence of **7** on the gels was imaged on a Syngene scanner. The image in Fig 4 was modified in Adobe Photoshop from the original in the following ways: 1) green and red photo filters were applied to corresponding monochrome images for clarity; 2) the HEX image from the Typhoon scanner (green) was scaled to same size as the **7** image from the Syngene G:Box Chemi XT4 scanner (red) to account for different image resolutions and to facilitate co-registration; 3) a levels adjustment filter was applied uniformly to the red image to increase signal-to-noise and improve contrast; 4) the images were cropped to the region of interest; and 5) a "screen" blending mode was applied to the green layer to allow the red layer to be observed beneath it without changing opacity settings. No quantitative measurements were taken from the modified images.

#### SSB activity assay control reactions

Samples were prepared in triplicate. To a 0.6 mL Eppendorf tube were added dsDNA (10 μL, 5 pmol), 10X UDG reaction buffer (1 μL), 10X APE reaction buffer (1 μL), H_2_O (5 μL), **7** or vehicle control (2 μL, 1 nmol; vehicle = 1% DMSO in H_2_O), and UDG or UDG storage buffer (1 μL, 5 Units). Samples were incubated at 37°C for 1 h in the dark. Then APE (1 μL, 10 Units) or APE storage buffer (1 μL) was added and samples were again incubated at 37°C for 1 h in the dark.

#### SSB activity assay with base pairs

Reaction samples were prepared in triplicate; control samples were prepared singly. To a 0.6 mL Eppendorf tube were added dsDNA (10 μL, 5 pmol), 10X UDG reaction buffer (1 μL), 10X APE reaction buffer (1 μL), H_2_O (5 μL), **7** or vehicle control (2 μL, 1 nmol; vehicle = 1% DMSO in H_2_O), and UDG (1 μL, 5 Units). Samples were incubated at 37°C for 10 min, 30 min, 1 h, 2 h, or 3 h in the dark. Then APE (1 μL, 10 Units) was added and samples were again incubated at 37°C for 1 h in the dark.

#### SSB activity assay UDG inhibition

Method UDG (10 min) +7: All samples were prepared in triplicate. To a 0.6 mL Eppendorf tube were added dsDNA (U:A, 10 μL, 5 pmol), 10X UDG reaction buffer (1 μL), 10X APE reaction buffer (1 μL), H_2_O (5 μL), and UDG (1 μL, 5 Units). Samples were incubated at 37°C for 10 min. Compound **7** (2 μL, 1 nmol) was added and the samples were incubated at 37°C in the dark for 2 min, 10 min, 30 min, 60 min, or 180 min. Then APE (1 μL, 10 Units) was added and samples were again incubated at 37°C for 1 h in the dark.

Method UDG+7: All samples were prepared in triplicate. To a 0.6 mL Eppendorf tube were added dsDNA (U:A, 10 μL, 5 pmol), 10X UDG reaction buffer (1 μL), 10X APE reaction buffer (1 μL), H_2_O (5 μL), **7** (2 μL, 1 nmol), and UDG (1 μL, 5 Units). Samples were incubated at 37°C in the dark for 2 min, 10 min, 30 min, 60 min, or 180 min. Then APE (1 μL, 10 Units) was added and samples were again incubated at 37°C for 1 h in the dark.

Method UDG: As in method UDG+**7** except substitute DMSO (2 μL) for **7**.

#### SSB activity assay APE inhibition

APE (2 μL, 20 Units) and **7** or vehicle (4 μL, 2 nmol; vehicle = 1% DMSO in H_2_O) were mixed and incubated at 37°C for 0.5 h, 1 h, 1.5 h, 2 h, or 3 h in the dark. Meanwhile, five samples of each DNA reaction were prepared. To a 0.6 mL Eppendorf tube were added dsDNA (U:A, 10 μL, 5 pmol), 10X UDG reaction buffer (1 μL), 10X APE reaction buffer (1 μL), H_2_O (5 μL), and UDG (1 μL, 5 Units). Samples were incubated at 37°C for 1 h in the dark. Then APE/**7** mixtures (3 μL) were added and samples were again incubated at 37°C for 1 h in the dark.

#### SSB activity assay ARP and MX comparison

Method 1: Stock solutions of probes were prepared at 50 mM. Compound **7** was dissolved in DMSO, MX was dissolved in H_2_O (pH 7), and ARP was dissolved in H_2_O. Stock solutions were further diluted with H_2_O to 500 μM. Water was used as a blank. All samples were prepared in triplicate. To a 0.6 mL Eppendorf tube were added dsDNA (U:A, 10 μL, 5 pmol), 10X UDG reaction buffer (1 μL), 10X APE reaction buffer (1 μL), H_2_O (5 μL), probe or blank (2 μL, 1 nmol), and UDG (1 μL, 5 Units). Samples were incubated at 37°C for 10 min, 30 min, 1 h, 2 h, or 3 h in the dark. Then APE (1 μL, 10 Units) was added and samples were again incubated at 37°C for 1 h in the dark.

Method 2: Stock solutions of 50 mM ARP and MX were used without further dilution. Compound **7** was diluted to 500 μM. APE was diluted 10-fold in APE storage buffer. Water was used as a blank. All samples were prepared in triplicate. To a 0.6 mL Eppendorf tube were added dsDNA (U:A, 10 μL, 5 pmol), 10X UDG reaction buffer (1 μL), 10X APE reaction buffer (1 μL), H_2_O (5 μL), probe or blank (4 μL; 2 nmol **7**, 200 nmol ARP and MX), and UDG (1 μL, 5 Units). Samples were incubated at 37°C for 10 min, 30 min, 1 h, 2 h, or 3 h in the dark. Then APE (1 μL, 1 Unit) was added and samples were again incubated at 37°C for 1 h in the dark.

### Heat/acid treatment of calf thymus DNA

Calf thymus DNA was purchased from Sigma and was reconstituted overnight at 4°C in either H_2_O or 500 mM MX (to eliminate basal AP sites) with gentle shaking (1.5 mL water or solution per 5 mg DNA). For samples reconstituted in MX solution, an ethanol precipitation was performed twice before heat and acid treatment (*vide infra*) then reconstituted in water. The DNA-water solution was aliquoted to 1.5 mL Eppendorf tubes (360 μL) and 10X Citrate buffer (1M NaCl, 100 μM monosodium phosphate, 100 μM monosodium citrate, pH 5.0) was added to a final concentration of 1X. For t = 0 min samples, ice-cold 100% EtOH (1.0 mL) was added immediately and the Eppendorf tubes stored at -20°C. Other samples were placed in a 70°C heating block for 15–90 minutes in 15-minute increments. Samples were removed from heat, precipitated in ice-cold 100% EtOH (1.0 mL), and then chilled at -20°C for at least 20 minutes. Samples were centrifuged at 12,000 x G for 10 min. at 4°C. The supernatant was discarded. The process was repeated once beginning with the addition of EtOH (1.0 mL). The DNA pellet was dissolved in 700 μL H_2_O or TE buffer. Aliquots of 80 μL were put in 1.5 mL Eppendorf tubes. Any samples not used immediately were stored at -80°C.

#### Genomic DNA time course of 7 binding

A 100 mM stock solution of **7** in DMSO was diluted to 25 μM in H_2_O. Heat/acid treated DNA (t = 45 min, 80 μL) were warmed to 37°C. In triplicate, **7** (20 μL) was added to the DNA pipetting up and down to mix. Samples were kept at 37°C in the dark for the following incubation times (min): 0.5, 1, 2, 5, 10, 15, 30, and 60. Immediately after the incubation time, ice-cold EtOH (1.0 mL) was added. Samples were quickly inverted to mix then stored in the dark at -78°C in a dry ice-EtOH bath. The DNA was then purified by EtOH precipitation three times as described in the heat/acid treatment of genomic DNA. The purified DNA pellets were suspended in 150 μL H_2_O. Aliquots (125 μL) of the samples were added to a black, clear-bottom 96 well plate (Corning) and analyzed with 750 nm excitation and 800 nm emission. Fluorescence was adjusted to DNA concentration.

#### Genomic DNA dose-response of 7

Samples were prepared in triplicate. To 90 μL aliquots of heat/acid treated DNA (t = 20 min), was added **7** (10 μL) in the following quantities (pmol): 0, 0.05, 0.5, 5, 50, 250, 500, 2500, 5000, 12500, 25000, and 50000. Solutions were kept in the dark. Samples were incubated for 1 h at 37°C in the dark. Ice-cold EtOH (1.0 mL) was added and the DNA was purified by EtOH precipitation three times as described in the heat/acid treatment of genomic DNA section. DNA was dissolved in 120 μL H_2_O. Aliquots (100 μL) of the samples were added to a black, clear-bottom 96 well plate (Corning) and analyzed with 750 nm excitation and 800 nm emission. Fluorescence was adjusted to 300 μg/mL DNA concentration.

#### MX and 7 genomic DNA competition assay

Solutions of MX and **7** were prepared in H_2_O varying [MX] from 0–1.9 M and maintaining [7] at 25 μM. CAUTION: Solutions were kept vigorously in the dark as MX was observed to decompose **7** rapidly (on the minute time scale) in the presence of light. The ratios of MX:**7** used in this study were: 0, 0.01, 0.1, 0.5, 1, 5, 10, 50, 100, 250, 500, 1000, 2500, 5000, 10000, 25000, 50000, and 75000. For visual clarity, the data for the ratios 0.01, 0.1, 5, 10, 50, 250, and 500 were omitted from the Fig 12 as this did not affect the analysis. The following samples were prepared in triplicate: 20 μL of MX/**7** solution was added to 80 μL aliquots of heat/acid treated DNA (t = 45 min). Samples were incubated for 1 h at 37°C in the dark. Ice-cold EtOH (1.0 mL) was added and the DNA was purified by EtOH precipitation three times as described in the heat/acid treatment of genomic DNA section. DNA was dissolved in 150 μL H_2_O. Aliquots (125 μL) of the samples were added to a black, clear-bottom 96 well plate (Corning) and analyzed with 750 nm excitation and 800 nm emission. Fluorescence was adjusted to DNA concentration.

#### ARP and 7 genomic DNA competition assay

As before (section 2.3.2) except solutions of ARP and **7** were prepared in H_2_O varying [ARP] from 0–75 mM and maintaining [7] at 1 μM. The ratios of ARP:**7** used in this study were: 0, 0.1, 1, 10, 100, 500, 1000, 5000, 10000, 25000, 50000, and 75000.

#### MX and 7 genomic DNA blocking assay

To 80 μL aliquots of DNA was added either 10 μL of MX (500 mM, pH 7) or vehicle control (500 mM NaCl). TE buffer was used as a-DNA control. Samples were incubated at 37°C for 30 min. Then, either 10 μL of **7** (500 μM) or 10 μL of 1%DMSO in water was added to samples. Samples were incubated at 37°C in the dark for 1 h before DNA was purified by ethanol precipitation three times as described in the heat/acid treatment of genomic DNA. The purified DNA pellets were dissolved in 150 μL H_2_O. Aliquots (125 μL) of the samples were added to a black, clear-bottom 96 well plate (Corning) and analyzed with 700 nm excitation and 790 nm emission. Fluorescence was adjusted to DNA concentration.

### AP site evaluation in FUDR treated cells

#### Preparation of KD and WT cells

DLD1 cells (ATCC #CCL-221) were obtained from the laboratory of Sanford Markowitz at Case Western Reserve University. UDG directed shRNA clones and scrambled targeted control shRNA clones were purchased from Sigma-Aldrich. According to manufacturer’s instructions from Lipofectamine 2000 (Invitrogen), HEK293 cells were transfected to produce lentiviral particles that were used to infect DLD1 cells. Forty-eight hours after transfection, DLD1 cells were diluted for passage and selected with puromycin. The UDG knockdown levels were verified by RT-PCR and Western blot analysis.

#### FUDR Treatment, isolation, and compound 7 analysis of DLD1 DNA

DLD1 shUDG (KD) or shControl (WT) cells were plated in Falcon brand 100x20 mm cell culture dishes in 10 mL medium (DMEM supplemented with 10% heat inactivated FBS, penicillin/streptomycin, and nonessential amino acids) and incubated at least 16 h at 37°C and 5% CO_2_ to ensure adhesion. To allow for cell proliferation, the following approximate numbers of cells were plated for each time point given in parenthesis: 4 million (24 h), 1 million (48 h), and 0.3 million (72 h). Two mL of medium were removed from each plate and replaced with 2 mL of FUDR solution at a final concentration of 0, 10, 200, or 1000 nM in 10 mL. For each time point, six plates of each FUDR treatment group were prepared for each cell line. Cells were incubated at 37°C and 5% CO_2_ with continuous FUDR exposure for 24, 48, or 72 h.

At the time points, the media was removed and cells were rinsed with PBS. Cells were dissociated with 0.25% trypsin (1 mL) and transferred to 15 mL conical tubes in 5 mL PBS. The conical tubes were centrifuged at 1,700 rpm to pellet the cells. The supernatant was removed and discarded. Cell pellets were dissolved in TE buffer (2 mL) then treated with 10% SDS (240 μL) and RNase A (10 μL, 20 mg/mL purchased from Invitrogen) for at least 15 minutes at 37°C. Then, proteinase K (10 μL, 20 mg/mL purchased from Invitrogen) was added and cell lysates were incubated for a least 15 minutes at 37°C. Cell lysates were transferred to Phase Lock Gel Light 15 mL conical tubes purchased from 5Prime. Saturated phenol (2 mL, pH 6.6) was added to the cell lysates and the mixture was shaken vigorously. Chloroform (0.5 mL) was then added and the cell lysate mixtures shaken vigorously. The organic and aqueous phases were separated by centrifuging the gel tubes at 2,000 rpm for 10 minutes. After a second round of phenol-chloroform addition and centrifugation, pure chloroform (2 mL) was added to the cell lysates, shaken, and centrifuged at 2,000 rpm for 10 minutes. The aqueous layer containing the isolated DNA was decanted into a clean 15 mL conical tube and precipitated with 100% EtOH (5 mL) and 3M sodium acetate (100 μL) by gentle rocking at 4°C for at least 30 minutes. DNA was isolated by centrifuging at 3,000 rpm for 10 minutes. The DNA pellets were washed once with 70% EtOH (1.5 mL) and centrifuged at 3,000 rpm for 10 minutes.

Pure DNA pellets were dissolved in 200 μL 1X UDG buffer. Samples were treated in triplicate with either UDG (1 μL, 5 units) or UDG storage buffer (see SSB activity assay procedures) and incubated at 37°C for 1 h. After UDG incubation, 10 μL of each solution was removed and set aside for analysis with the ARP assay (Dojindo). A solution of **7** (10 μL, final = 25 μM) was added to each sample, keeping the stock solution and all treated samples vigorously in the dark until the final analysis. Samples were incubated with **7** for 1 h at 37°C in the dark. After incubation, ice cold 100% EtOH (1 mL) and 3M sodium acetate (5 μL) were added to each sample. Samples were inverted to mix then chilled for at least 20 minutes at -20°C. Samples were centrifuged at 12,000 G and 4°C for 10 minutes. The supernatant was discarded. This EtOH wash was repeated twice with 70% EtOH (1 mL) and no sodium acetate. DNA pellets were dissolved in H_2_O (minimum 150 μL). DNA concentrations were measured and adjusted to a maximum of 300 μg/mL. Aliquots (125 μL) of the samples were added to a black, clear-bottom 96 well plate (Corning) and analyzed with 760 nm excitation and emission scan of 790–847 nm with a 3 nm step size. Integrated fluorescence intensities were adjusted to DNA concentration and normalized to the FUDR untreated KD +UDG sample.

#### ARP analysis of FUDR treated DLD1 DNA

DNA aliquots (10 μL) from above were diluted in TE buffer (10 μL). DNA concentrations were measured on a Nanodrop 1000 and adjusted to 100 μg/mL in TE buffer. Samples were prepared for ARP analysis using the ARP DNA Damage Quantification Kit (Dojindo #DK02-12). Samples were prepared and analyzed according to the manufacturer’s instructions.

#### MMS Treatment, isolation, and compound 7 analysis of DLD1 DNA

DLD1 shControl (WT) cells at a density of 3 million cells per dish were plated in Falcon brand 100x20 mm cell culture dishes in 10 mL medium (DMEM supplemented with 10% heat inactivated FBS, penicillin/streptomycin, and nonessential amino acids) and incubated 16 h at 37°C and 5% CO_2_ to ensure adhesion. Solutions of MMS were prepared in serum free DMEM medium at concentrations of 0.05, 0.25, 1, 4, and 10 mM. Medium was removed from the cells and replaced with the MMS or a serum free medium vehicle. Three plates were prepared per treatment group. Cells were incubated at 37°C and 5% CO_2_ with continuous MMS exposure for 3 h. At the time points, cells were collected and DNA isolated as described above (Treatment of DLD1 cells with FUDR).

Pure DNA pellets were suspended in 190 μL water. A solution of **7** (10 μL, final = 25 μM) was added to each sample, keeping the stock solution and all treated samples vigorously in the dark until the final analysis. Samples were incubated with **7** for 1 h at 37°C in the dark. After incubation, DNA was purified and analyzed as described above.

## Supporting Information

S1 Fig ARP analysis of AP sites in DNA isolated from DLD1 cells. (TIF)

S2 Fig Side-by-side comparison of ARP and 7 assays. (TIF)

S3 Fig Analysis of APE inhibition with THF substrate. (TIF)

S1 File Additional experimental details and NMR spectra. (DOCX)

S2 File Raw electrophoresis data for main text Figs 4–8 and 13. (DOCX)
