# Supplementary figures and images for: A Fluorescent Probe to Measure DNA Damage and Repair
Source: PLoS One. 2015 Aug 26;10(8):e0131330. doi: 10.1371/journal.pone.0131330 (PMC4550365; doi:10.1371/journal.pone.0131330)

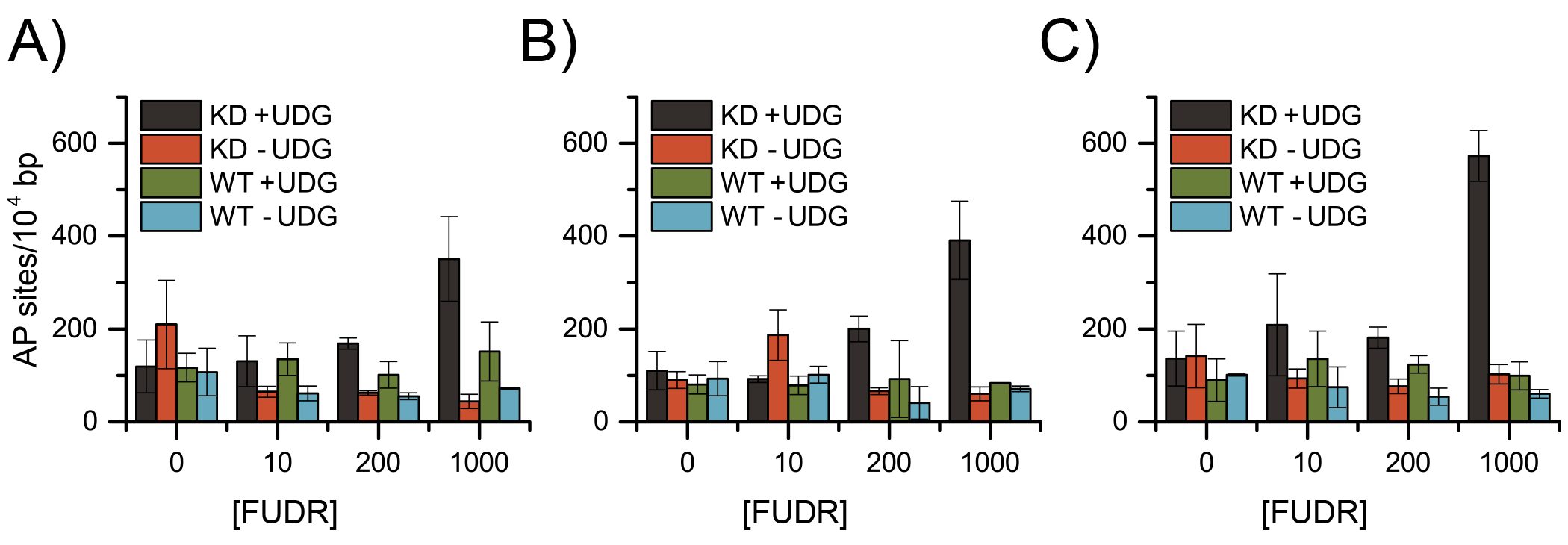

Supplement: S1 Fig — (TIF) [file pone.0131330.s001.tif]

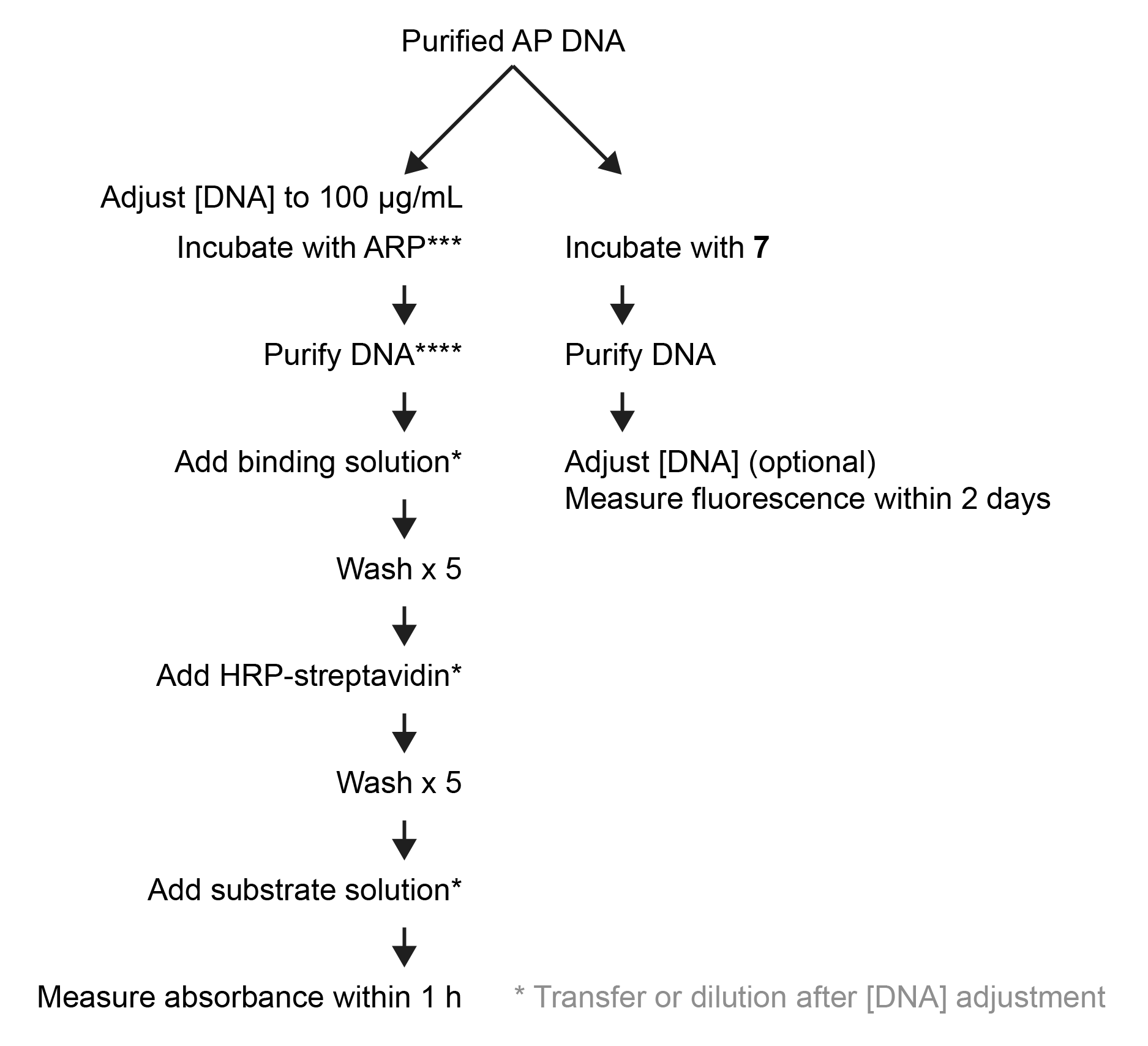

Supplement: S2 Fig — (TIF) [file pone.0131330.s002.tif]

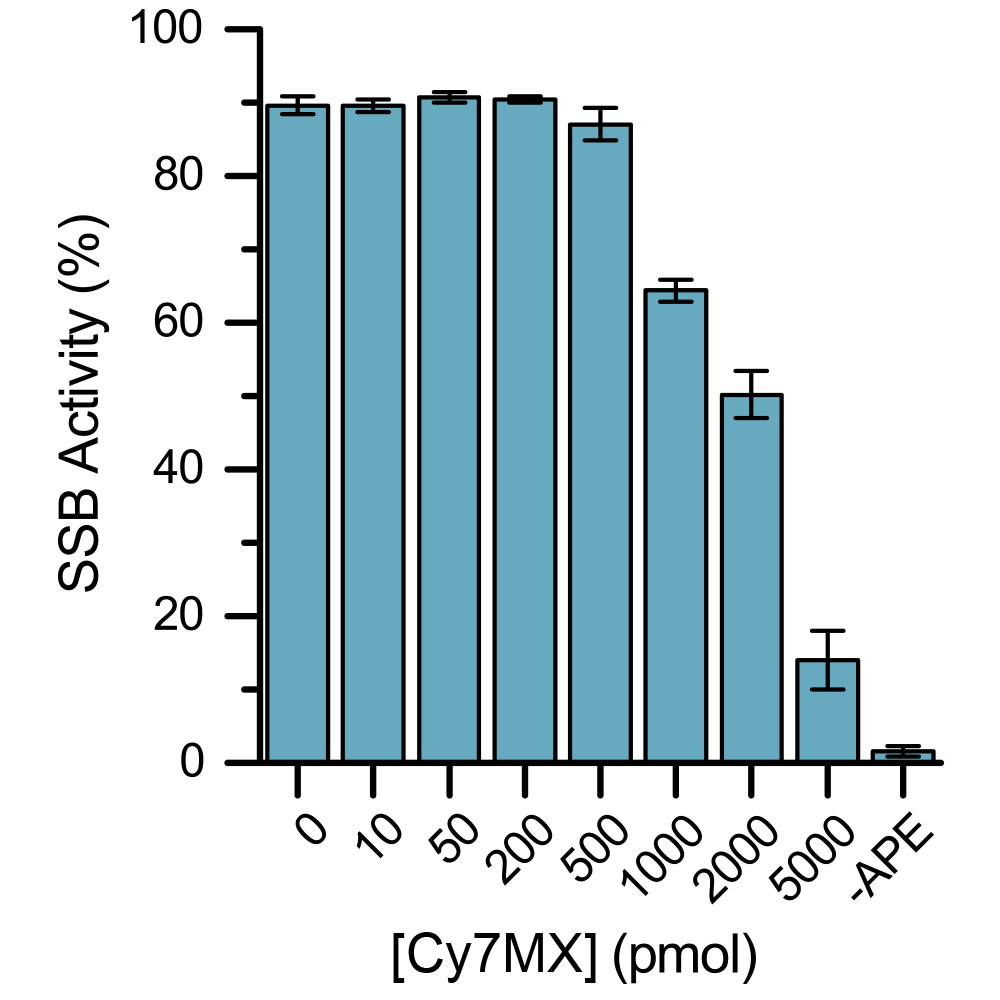

Supplement: S3 Fig — (TIF) [file pone.0131330.s003.tif]
