# Supplementary material for: A Fluorescent Probe to Measure DNA Damage and Repair
Source: PLoS One. 2015 Aug 26;10(8):e0131330. doi: 10.1371/journal.pone.0131330 (PMC4550365; doi:10.1371/journal.pone.0131330)

**Supporting Information for**

A Fluorescent Probe to Measure DNA Damage and Repair

Allison G. Condie,^1^ Yan Yan,^2^ Stanton L. Gerson,^3^ and Yanming Wang^1*^

^1^ Division of Radiopharmaceutical Science, Case Center for Imaging Research, Department of Radiology, Chemistry, and Biomedical Engineering, Case Western Reserve University, Cleveland, Ohio, United States

^2^ Department of Pharmacology, Case Western Reserve University, Cleveland, OH, United States

^3^ Department of Hematology and Oncology, Case Comprehensive Cancer Center, Case Western Reserve University, Cleveland, OH, United States

^*^ Correspondence should be addressed to Y.W. (E-mail): [yxw91@case.edu](mailto:yxw91@case.edu); (tel) +1-216-844-3288; (fax) +1-216-844-8062.

Table of Contents:

1. [Abbreviations 2](#_Toc417046213)
2. [Materials 3](#_Toc417046214)
3. [Detailed reaction procedures. 3](#_Toc417046215)
4. [Fluorescence quantum yield measurements. 4](#_Toc417046216)
5. [Evaluation of APE inhibition on THF substrate. 4](#_Toc417046217)
6. [References. 5](#_Toc417046218)
7. [^1^H and ^13^C NMR of all new compounds 6](#_Toc417046219)

## Abbreviations

AP: apurinic/apyrimidinic

APE: AP endonuclease

APS: ammonium persulfate

ARP: aldehyde reactive probe

BSA: bovine serum albumin

DBU: 1,8-diazabicyclo[5.4.0]undec-7-ene

DCM: dichloromethane

DMF: dimethylformamide

DMSO: dimethylsulfoxide

dsDNA: double stranded DNA

DTT: dithiothreitol

EDC: *N*-(3-dimethylaminopropyl)-*N*′-ethylcarbodiimide

EDTA: ethylenediaminetetraacetate

ESI: electrospray ionization

EtOAc: ethyl acetate

EtOH: ethanol

FBS: fetal bovine serum

Φ: fluorescence quantum yield

FUDR: 5-fluoro-2′-deoxyuridine

HEX: hexachlorofluorescein

HPLC: high pressure liquid chromatography

HRMS: high resolution mass spectrometry

HOBt: 1-​hydroxy-1*H*-​benzotriazole

ICG: indocyanine green or IR-125

MeCN: acetonitrile

MeOH: methanol

MX: methoxyamine

PAGE: polyacrylamide gel electrophoresis

PBS: phosphate buffered saline

SSB: single strand break

ssDNA: single stranded DNA

TBE: tris-borate-EDTA buffer

TE: tris-EDTA buffer

TEMED: tetramethylethylenediamine

TFA: trifluoroacetic acid

TLC: thin layer chromatography

TMS: tetramethylsilane

UDG: uracil DNA glycosylase

Materials. EDC·HCl was purchased from Advanced Asymmetrics. HOBt·H_2_O was purchased from AnaSpec. DMF, laser grade ICG (IR-125), and spectroscopic grade EtOH were purchased from Acros (Fisher Scientific). HPLC grade acetonitrile, HPLC grade chloroform, and HPLC grade water were purchased from Fisher Scientific. *E. coli* Uracil-DNA gylcosylase (UDG) and human AP Endonuclease (APE 1) were purchased from New England BioLabs. Boc anhydride, *N*-(3-bromopropyl)phthalimide, *tert*-butyl hydroxycarbamate, and trifluoroacetic acid were purchased from Oakwood. Calf thymus DNA, 1,8-Diazabicyclo[5.4.0]undec-7-ene, IR 780 iodide, and sodium hydride were purchased from Sigma Aldrich. Hydrazine hydrate was purchased from TCI America. Chromatography solvents were ACS grade and purchased from Fisher Scientific unless otherwise stated. Saline was purchased from Baxter (Deerfield, IL).

Detailed reaction procedures.

***tert*-butyl 3-(1,3-dioxoisoindolin-2-yl)propoxycarbamate (2)**. **2** was prepared according to a literature procedure.^1^ Briefly, *N*-(3-bromopropyl)phthalimide **1** (10.1 g, 37.5 mmol) and *tert*-butyl hydroxycarbamate (9.99 g, 75.0 mmol) were added to a dry 250 mL round bottom flask fitted with a stir bar. The flask was sealed with a rubber septum then the atmosphere was evacuated and refilled with argon 5 times. The reagents were dissolved in anhydrous DCM (60.0 mL), added via syringe. DBU was then added via a syringe and the reaction was stirred under argon at room temperature. After five hours, the reaction was quenched with 10% citric acid (50 mL) and extracted with DCM (3 x 30 mL). The combined organic layers were washed with 10% citric acid (2 x 50 mL), water (50 mL), then brine (50 mL). The organic layer was dried over MgSO_4_, filtered, and concentrated. The crude residue was diluted in a trace amount of DCM and purified by silica gel chromatography with a mobile phase of pure DCM then gradually increasing polarity to 9:1 DCM/EtOAc. Concentration gave **2** as a white solid (8.14 g, 68%). R_f_=0.45 (DCM/EtOAc, 9:1); ^1^H NMR (400 MHz, CDCl_3_): *δ*=7.85-7.81 (m, 2H), 7.73-7.68 (m, 2H), 7.34 (br s, 1H), 3.91 (t, J=6.4 Hz, 2H), 3.81 (t, J=6.7 Hz, 2H), 1.99 (tt, J=6.7, 6.4 Hz, 2H), 1.46 (s, 9H); ^13^C NMR (100 mHz, CDCl_3_): *δ*= 168.4, 156.8, 133.9, 132.0, 123.2, 81.7, 73.9, 35.0, 28.2, 27.2;

***tert*-butyl 3-aminopropoxycarbamate (3)**. **3** was prepared following modification of a literature procedure.^1^ Briefly, **2** (1.95 g, 6.10 mmol) was added to a 250 mL round bottom flask fitted with a magnetic stir bar. Methanol (100 mL) was added and the mixture was stirred until the solid was completely dissolved. Hydrazine hydrate (6.0 mL, 124 mmol, 1.032 g/mL) was added all at once while rapidly stirring. The reaction was stirred overnight at room temperature. The next morning, a white precipitate had formed. The methanol was removed in vacuo. The remaining residue was suspended in CHCl_3_ and filtered. The solid was washed several times with CHCl_3_ before the filtrate was transferred to a separatory funnel, diluted with water, and extracted. The water was extracted twice more with fresh CHCl_3_. The organic layers were combined and washed twice with water and once with brine. The organic layer was dried over Na_2_SO_4_, filtered, and concentrated to afford **3** as a pale yellow oil (1.02 g, 88%), which did not require further purification. ^1^H NMR (400 MHz, CDCl_3_): *δ*=3.95 (t, J=6.1 Hz, 2H), 2.85 (t, J=6.5 Hz, 2H), 1.77 (tt, J=6.5, 6.1 Hz, 2H), 1.47 (s, 9H); ^13^C NMR (100 mHz, CDCl_3_): *δ*= 156.8, 80.8, 74.3, 38.8, 31.0, 28.0;

## Fluorescence quantum yield measurements.


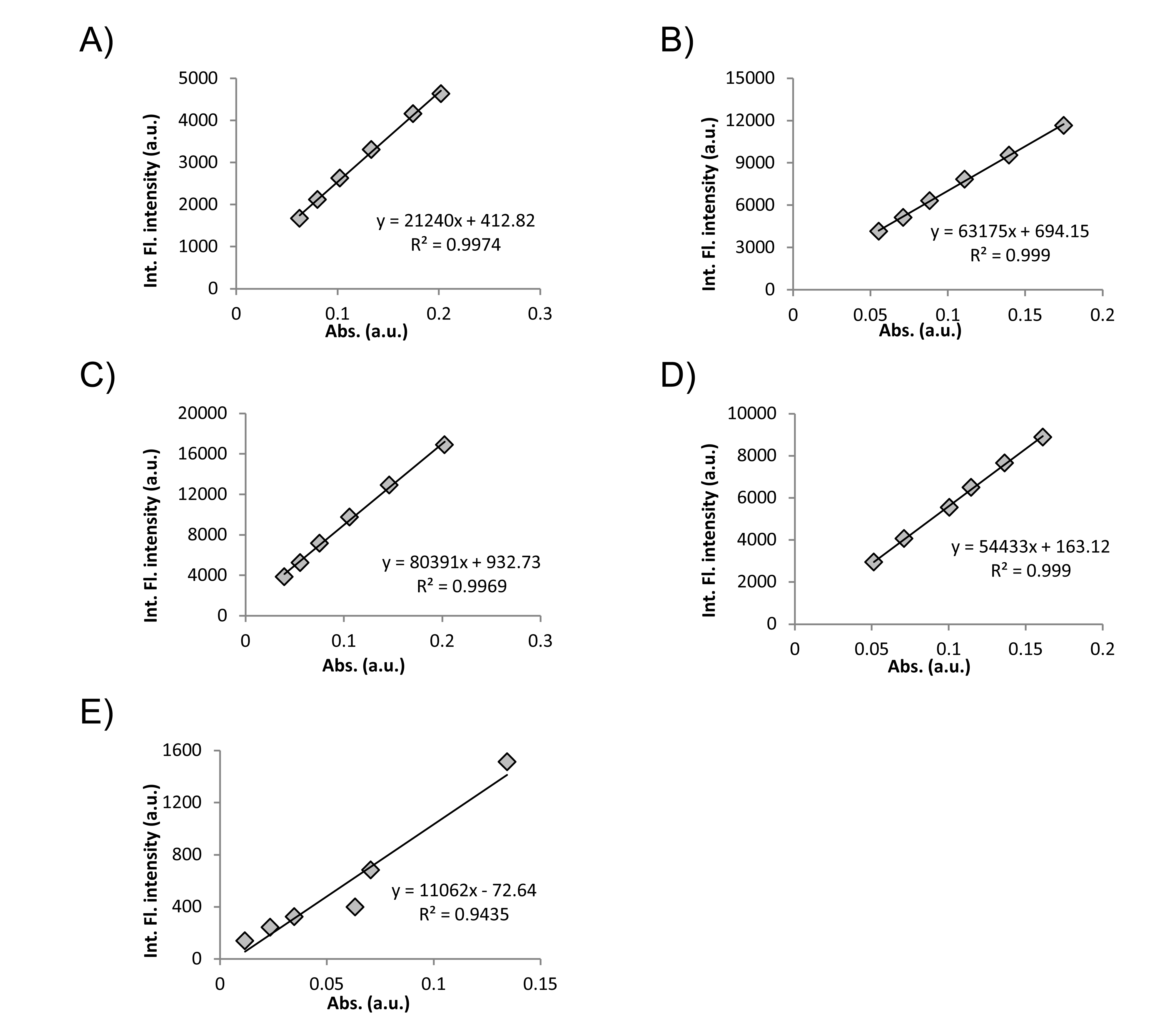


Figure S 1. To calculate m, integrated fluorescence intensity is plotted as a function of absorbance maxima for A) ICG in EtOH, B) **7** in EtOH, C) **7** in MeCN, D) **7** in CHCl_3_, and E) **7** in H_2_O.

## Evaluation of APE inhibition on THF substrate.

A second method was also used to detect APE inhibition. Tetrahydrofuran (THF) is an analog to the AP site that APE recognizes as a substrate but the AP site probes will not bind. A dsDNA oligomer with a THF:A base pair instead of a U:A base pair was used to evaluate if Cy7MX could inhibit APE directly without a background competition. A dose-response of **7** with a constant [APE] was observed for 1 h.

The data indicate that **7** has some inhibitory activity on the ability of APE to excise THF beginning at ~500 pmol. Except where otherwise noted, experiments were conducted using 1000 pmol of probe, and this corresponds to a 25% reduction in SSB activity for this system (S5 Fig.). The experiment suggests that 500 pmol would have been a better dose than 1000 nmol.

However, one should note that the THF:A substrate behaved differently than the U:A substrate. In the absence of **7**, the 90% SSB activity in the THF substrate is ca. 5% lower than what was observed in the AP site model (see Figs. 3-6). In addition, for the THF substrate with 1000 pmol of **7**, a 65% SSB activity is observed (a 25% reduction from the baseline, see figure below), whereas under the same conditions for the dU:A substrate, there was only a 10% reduction from baseline (Fig. 4). The competition of **7** with APE would be expected to make the dU:A SSB activity lower than the THF:A under similar conditions. These differences suggest that APE has different affinities for the two substrates and the extent of APE inhibition by Cy7MX may be contingent on the affinity.

SSB activity assays were performed on a 40-mer duplex DNA synthesized by Gene Link with the sequence:

5’-[HEX] TCCTGGGTGACAAAGCXAAACACTGTCTCCAAAAAAAATT

3’-AGGACCCACTGTTTCGYTTTGTGACAGAGGTTTTTTTTAA

where X=THF and Y=adenine. Three samples of each DNA reaction were prepared. To a 0.6 mL Eppendorf tube were added HEX-labeled dsDNA (THF:A, 10 μL, 5 pmol), 10X APE reaction buffer (2 μL), H_2_O (5 μL), and **7** (2 μL, 10, 50, 200, 500, 1000, 2000, or 5000 pmol) or a vehicle control. APE (1 μL, 10 Units) or APE storage buffer (1 μL) was added and aamples were incubated at 37 °C for 1 h in the dark. Loading dye (5 μL) was added to each sample then 10 μL of each sample was loaded onto a 1.0 mm thick, 10-well gel.

## References.

1. Salisbury, C.M.; Maly, D.J.; Ellman, J.A. Peptide Microarrays for the Determination of Protease Substrate Specificity. *J. Am. Chem. Soc.* **2002**, *124*, 14868-14870.

^1^H and ^13^C NMR of all new compounds
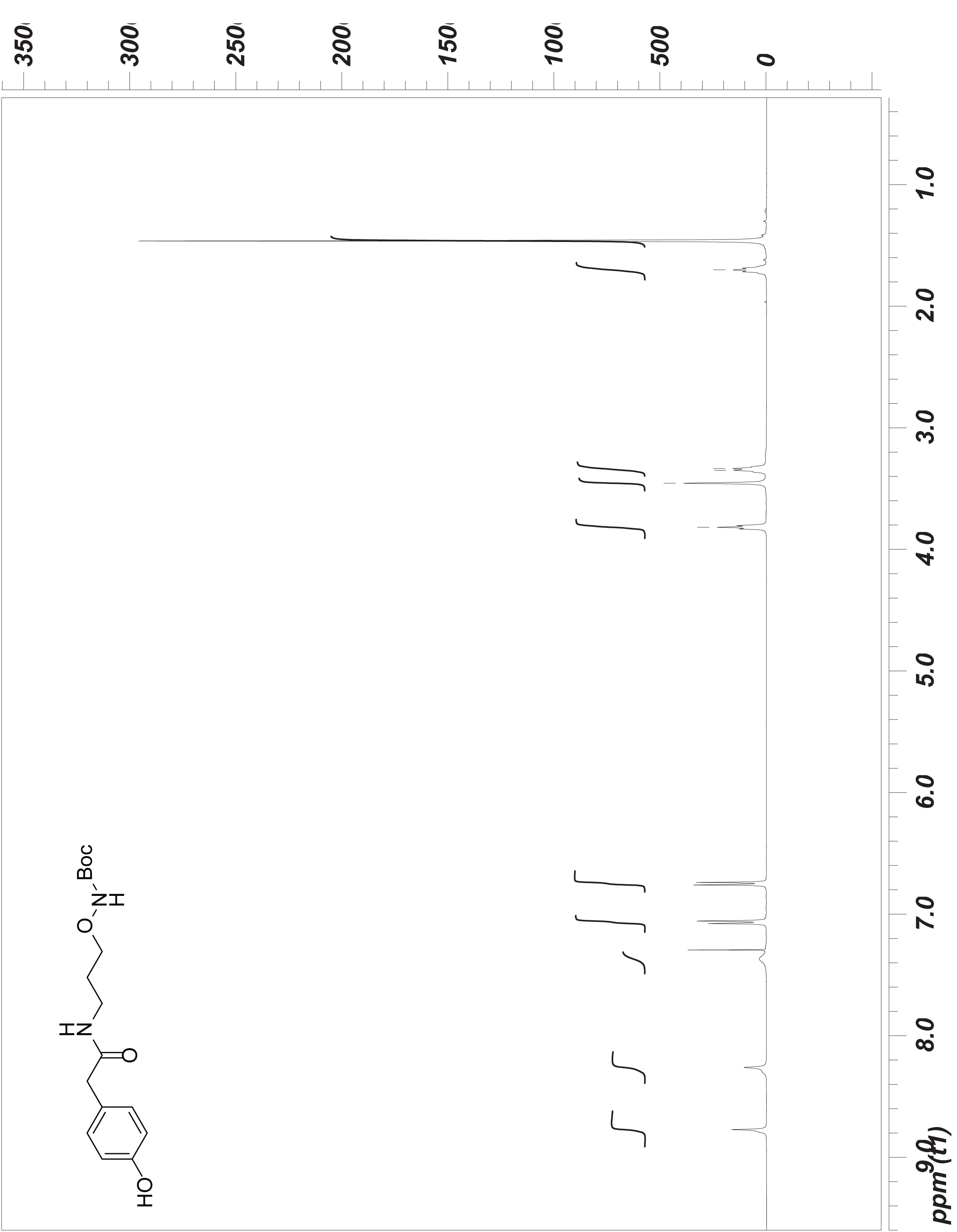


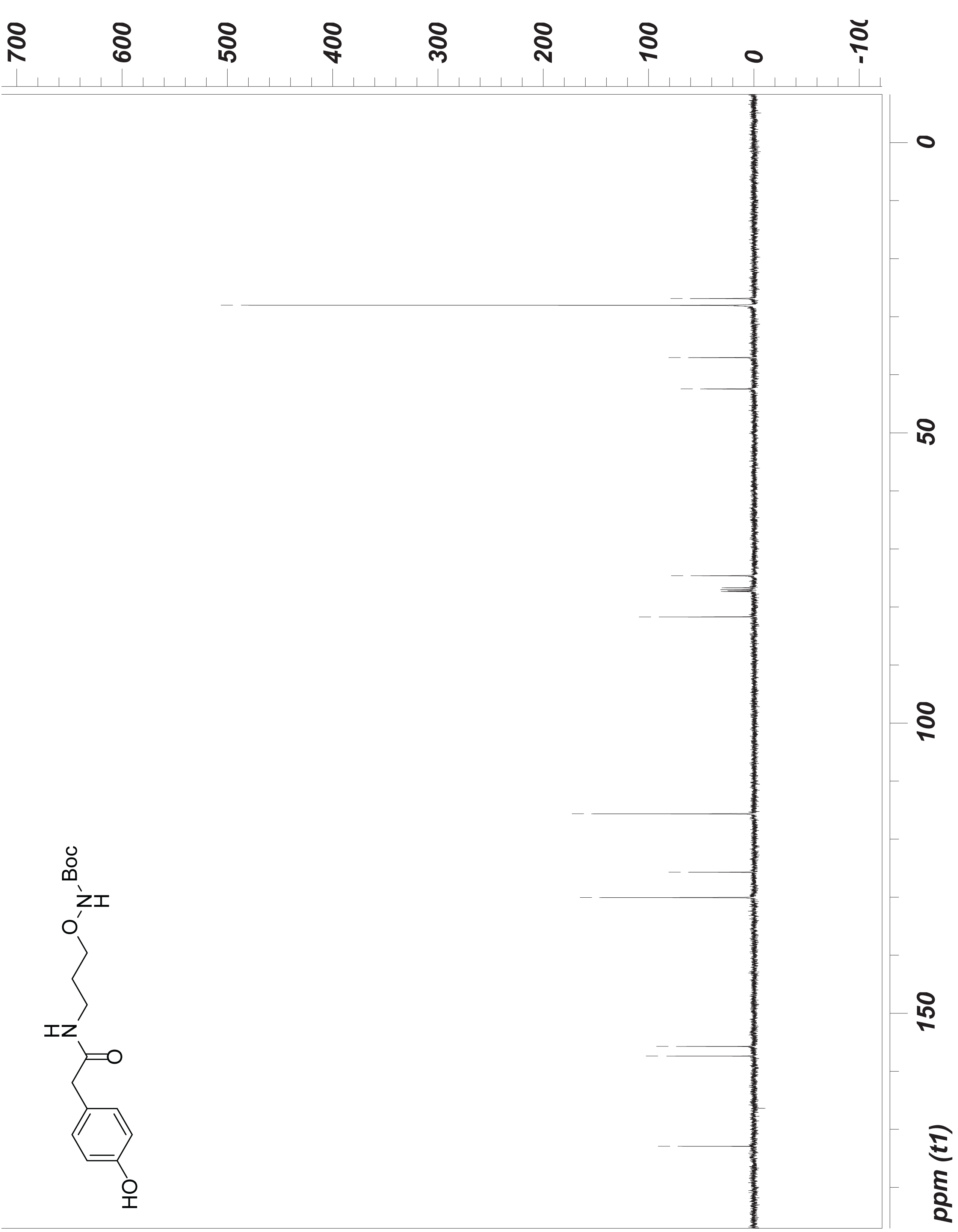


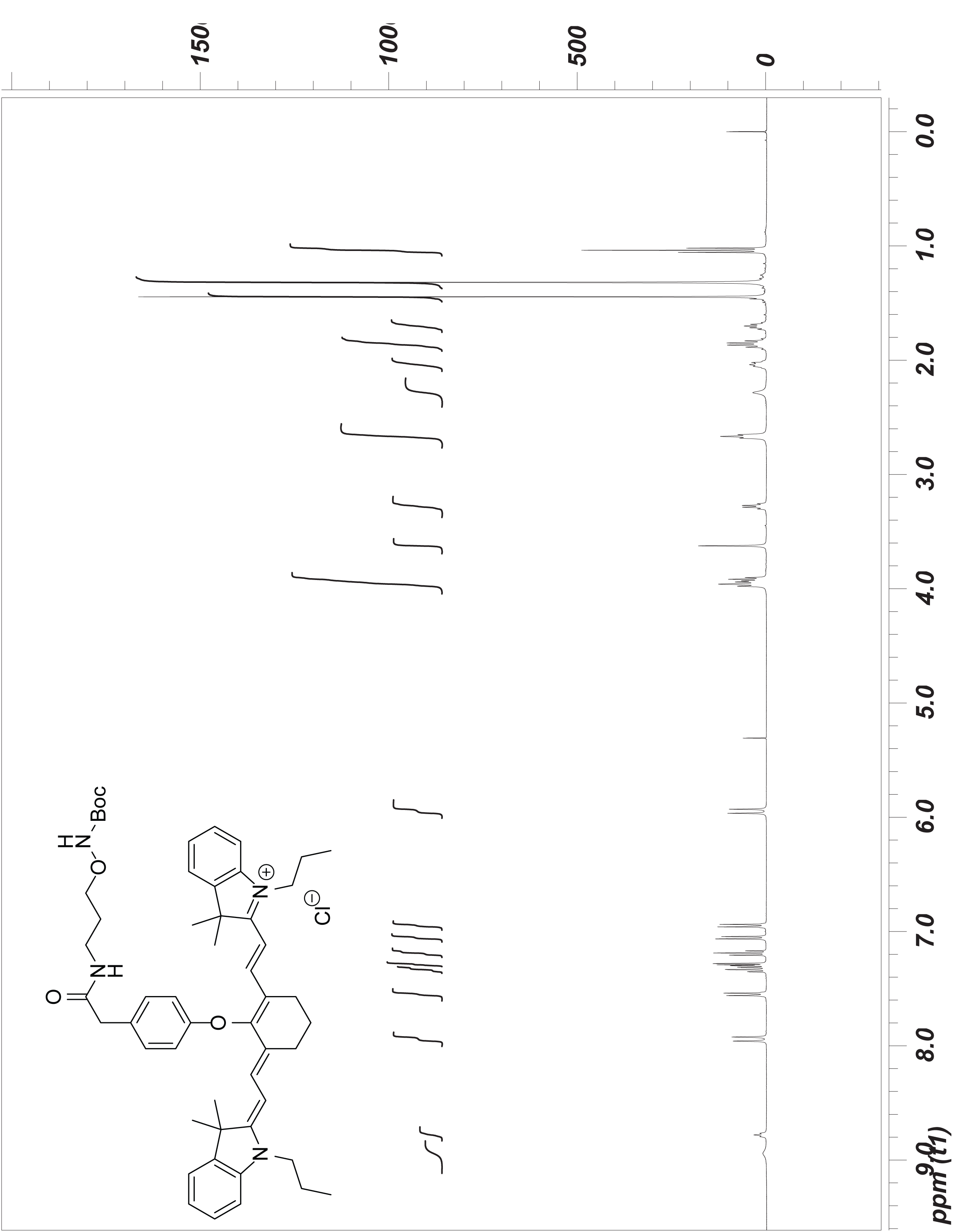


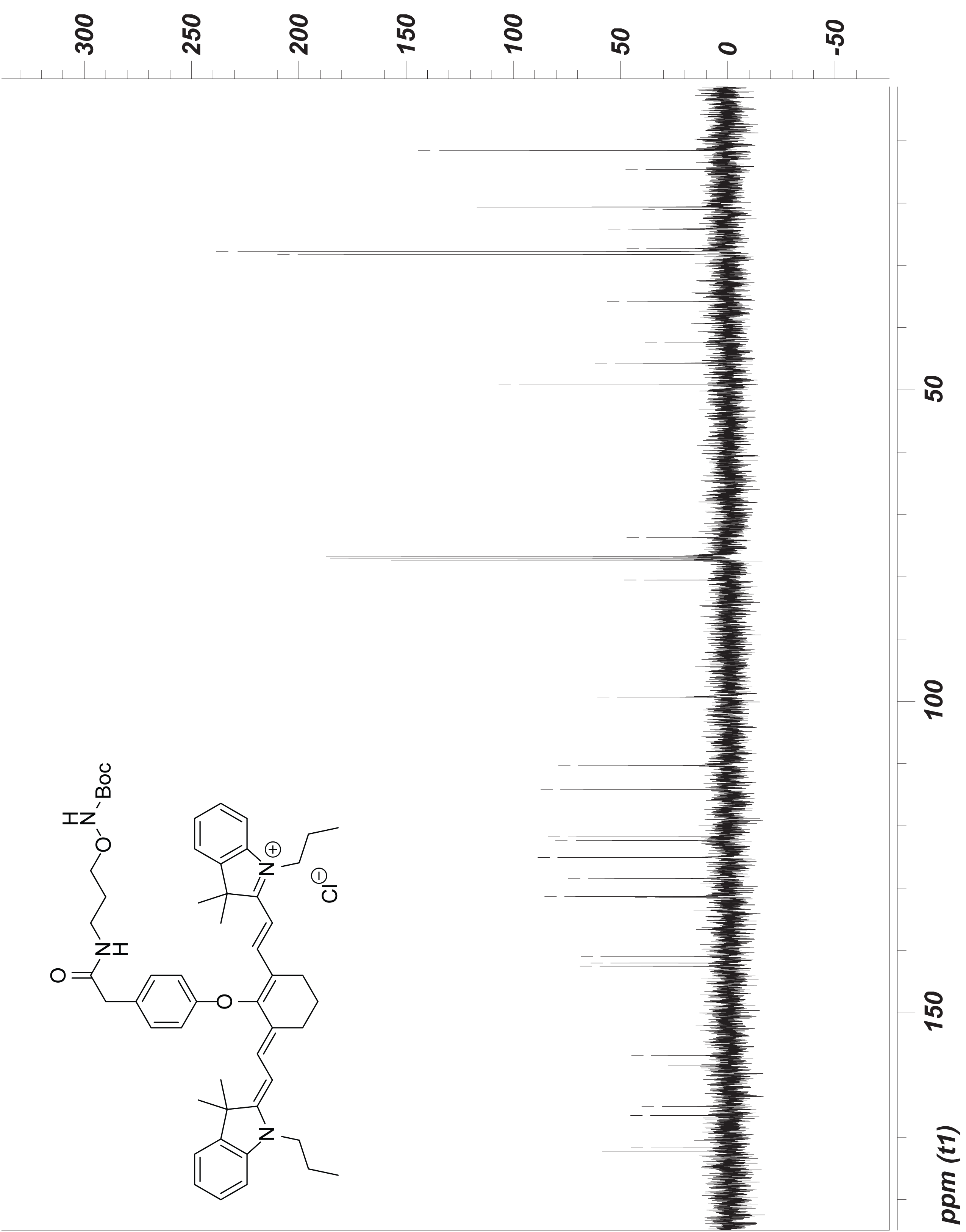


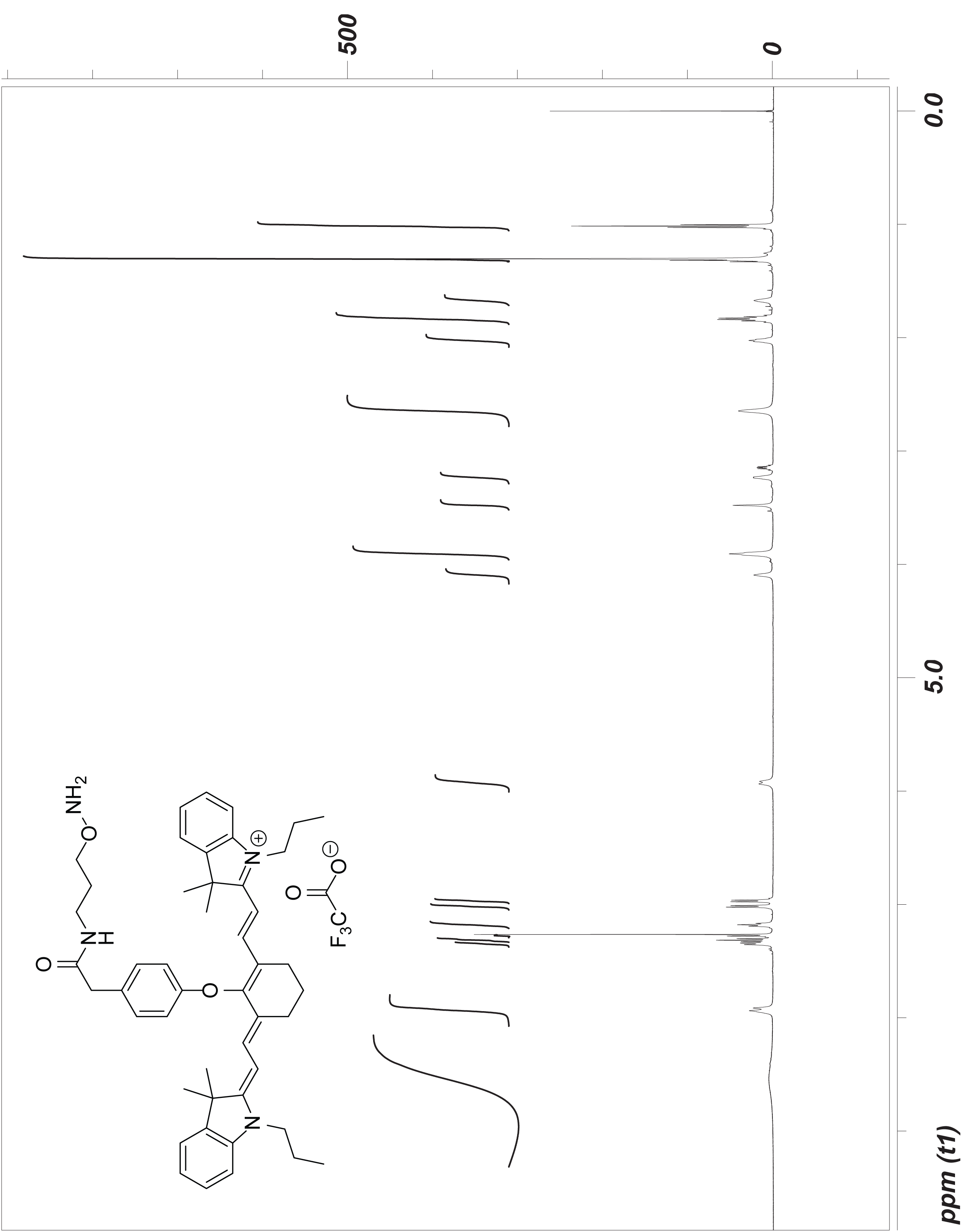


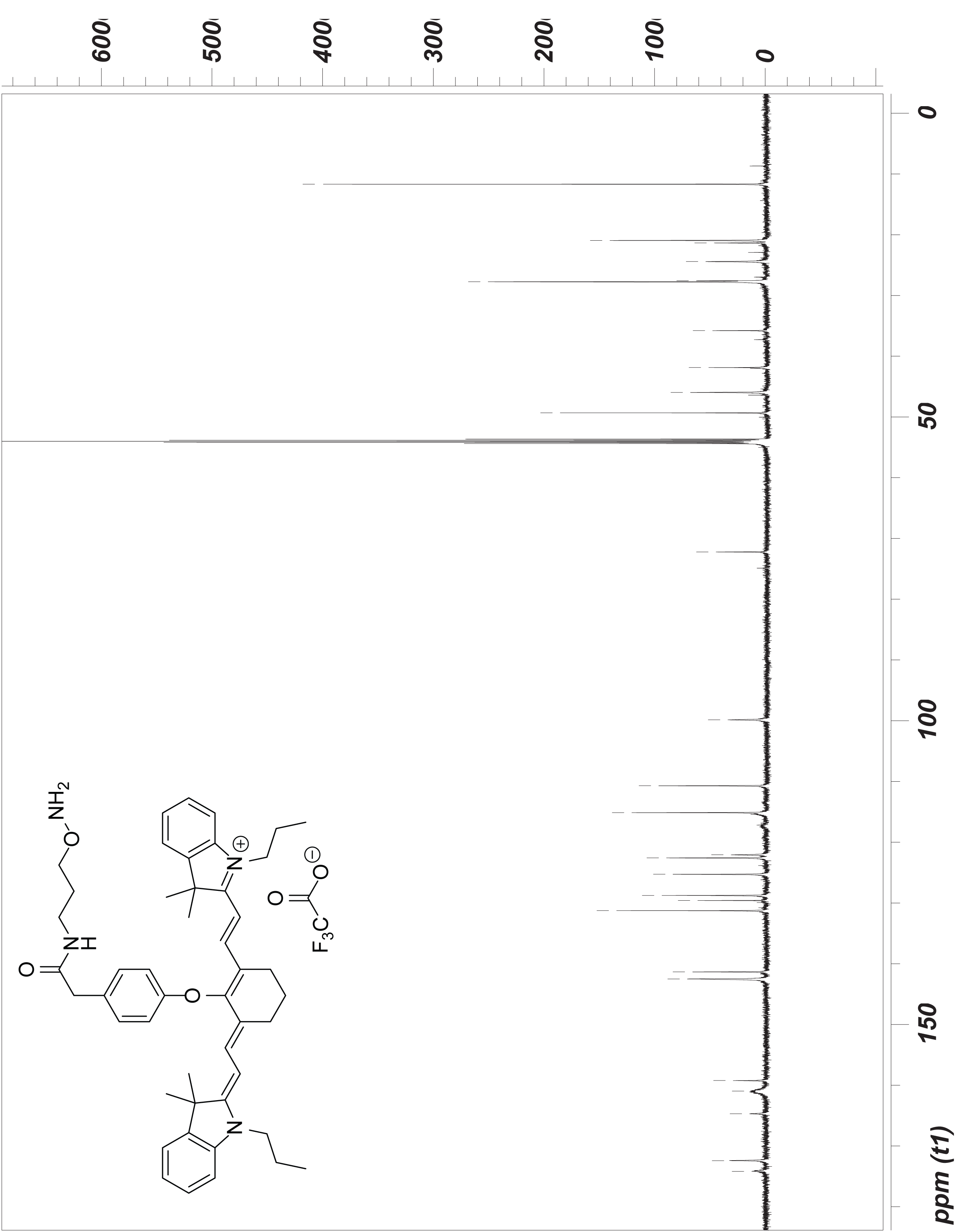

Supplement: S1 File — (DOCX) [file pone.0131330.s004.docx]
