# Supplementary material for: A Fluorescent Probe to Measure DNA Damage and Repair
Source: PLoS One. 2015 Aug 26;10(8):e0131330. doi: 10.1371/journal.pone.0131330 (PMC4550365; doi:10.1371/journal.pone.0131330)
Supplement: S2 File — (DOCX) [file pone.0131330.s005.docx]

**Supporting Information for**

A Fluorescent Probe to Measure DNA Damage and Repair

Allison G. Condie,^1^ Yan Yan,^1^ Stanton L. Gerson,^3^ and Yanming Wang^1*^

^1^ Division of Radiopharmaceutical Science, Case Center for Imaging Research, Department of Radiology, Chemistry, and Biomedical Engineering, Case Western Reserve University, Cleveland, Ohio, United States

^2^ Department of Pharmacology, Case Western Reserve University, Cleveland, OH, United States

^3^ Department of Hematology and Oncology, Case Comprehensive Cancer Center, Case Western Reserve University, Cleveland, OH, United States

^*^ Correspondence should be addressed to Y.W. (E-mail): [yxw91@case.edu](mailto:yxw91@case.edu); (tel) +1-216-844-3288; (fax) +1-216-844-8062.

Table of Contents:

1. [Gel electrophoresis for Figure 2. 2](#_Toc416695727)
2. [Gel electrophoresis for Figure 3. 4](#_Toc416695728)
3. [Gel electrophoresis for Figure 4. 5](#_Toc416695729)
4. [Gel electrophoresis for Figure 5. 6](#_Toc416695730)
5. [Gel electrophoresis for Figure 6. 7](#_Toc416695731)
6. [Western blot and q-RT-PCR analysis for Figure 11. 9](#_Toc416695732)

## Gel electrophoresis for Figure 2.


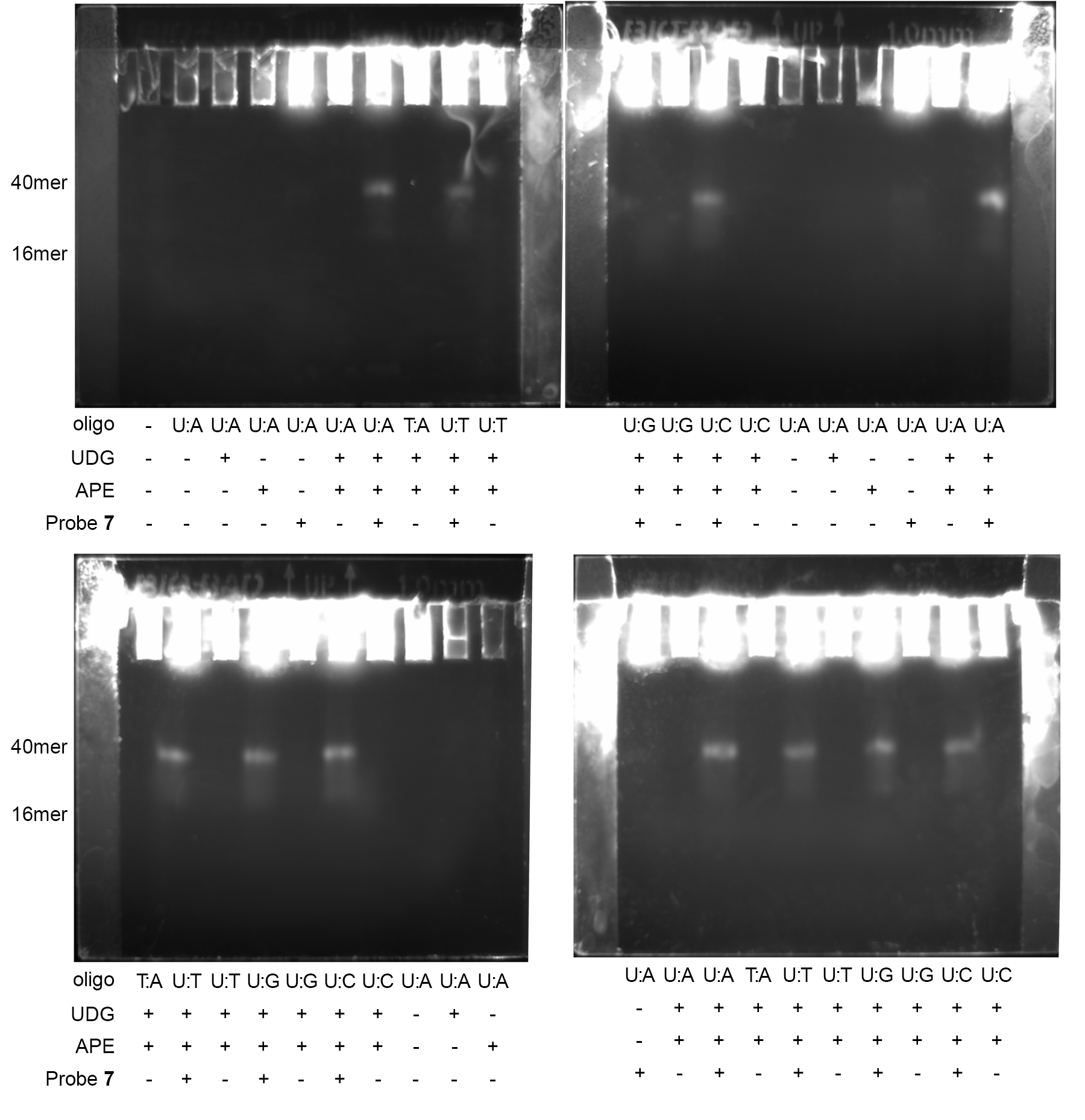


Figure S 1. Raw DNA denaturing PAGE (shown in monochrome scale) data used in qualification of compound **7** in Figure 2. Note that free **7** was not removed from the reaction, contains a positive charge, and travels opposite the direction of the DNA in the gel. This movement accounts for the intense brightness in wells containing or adjacent to samples where **7** is present.


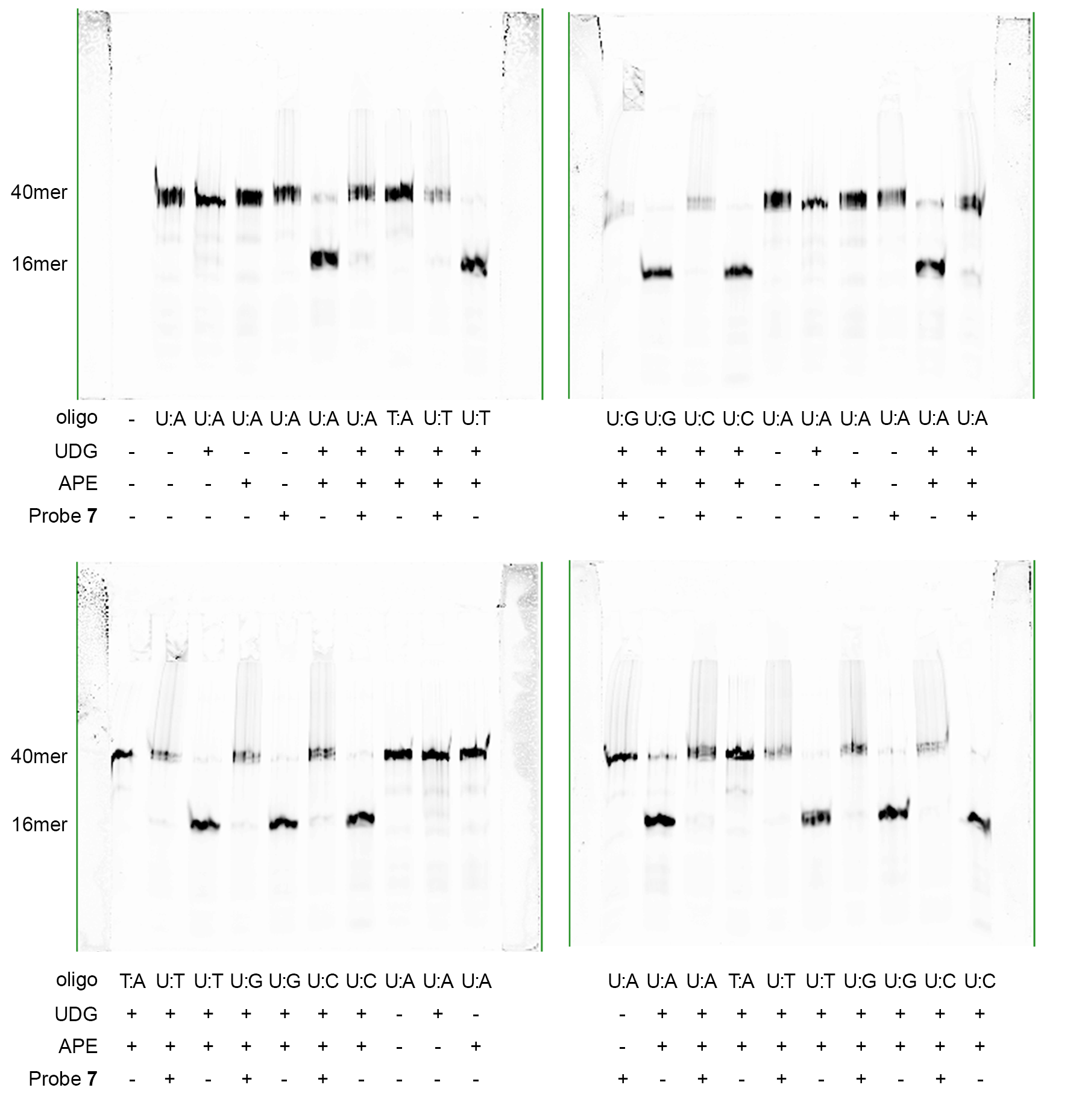


Figure S 2. Raw DNA denaturing PAGE (shown in grayscale) data used in qualification and quantification of HEX-labeled DNA in Figure 2.

Gel electrophoresis for Figure 3.
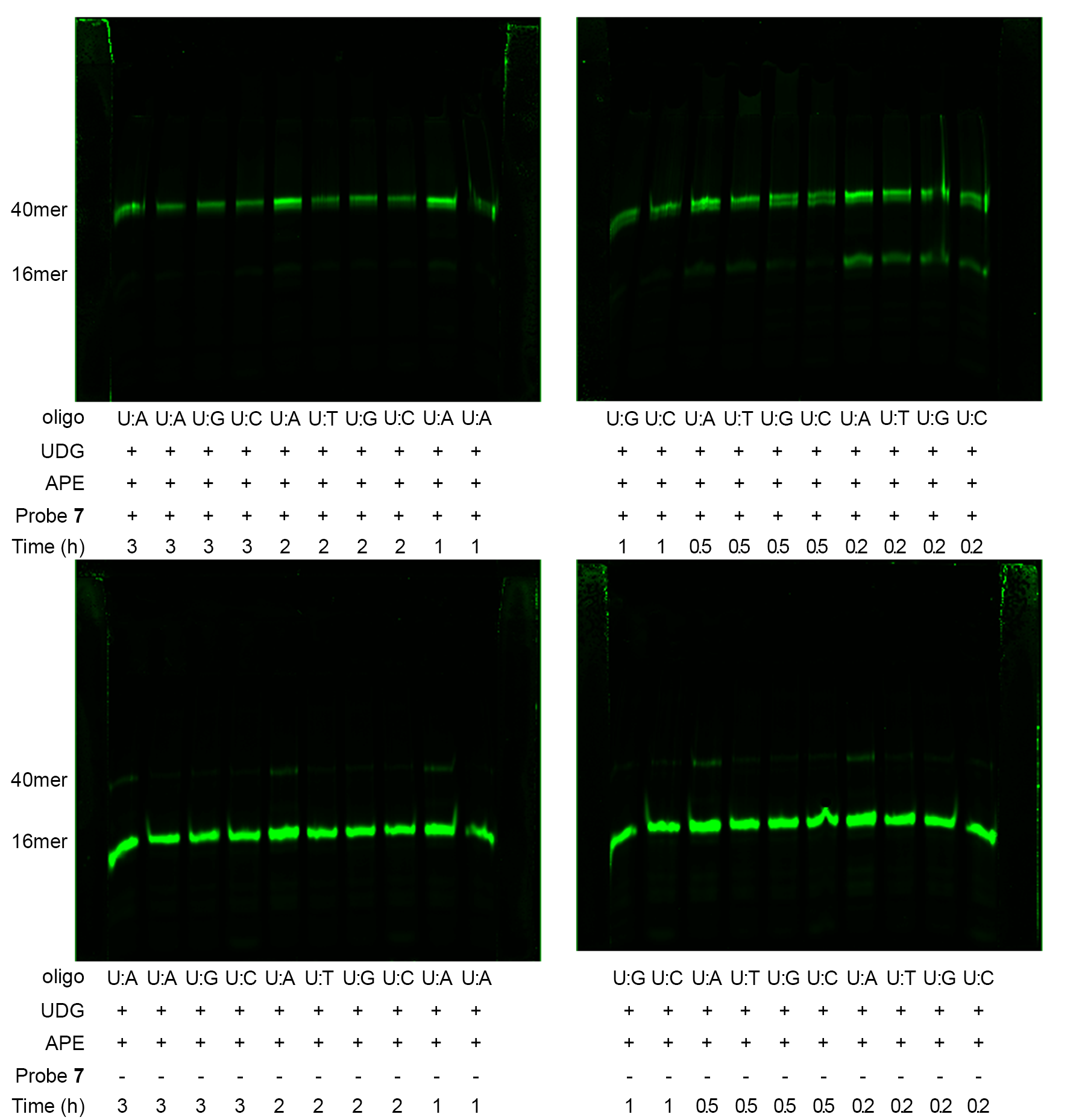


Figure S 3. Raw DNA denaturing PAGE (shown in greenscale) data used in quantification of HEX-labeled DNA in Figure 3.

## Gel electrophoresis for Figure 4.


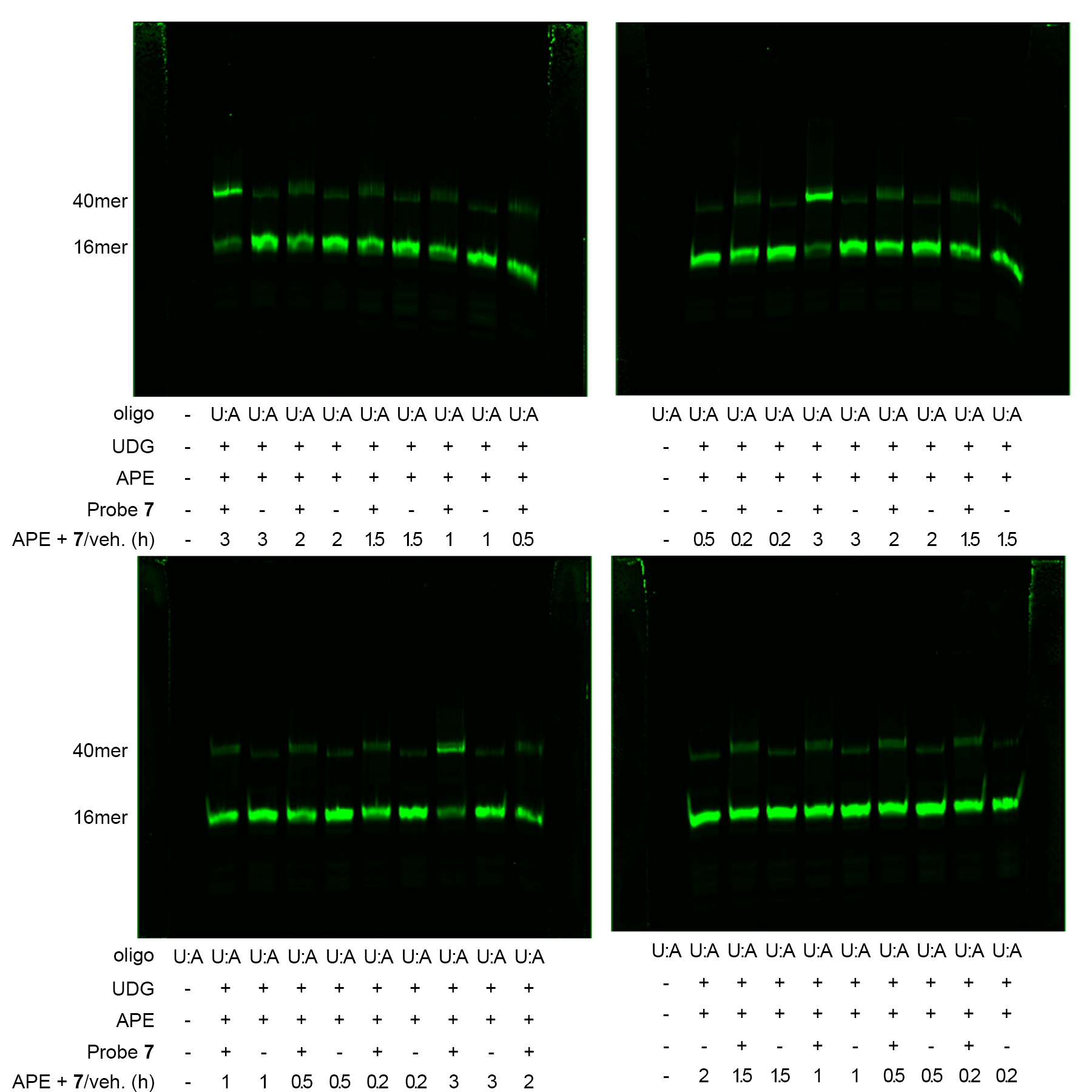


Figure S 4. Raw DNA denaturing PAGE (shown in greenscale) data used in quantification of HEX-labeled DNA in Figure 4.

## Gel electrophoresis for Figure 5.


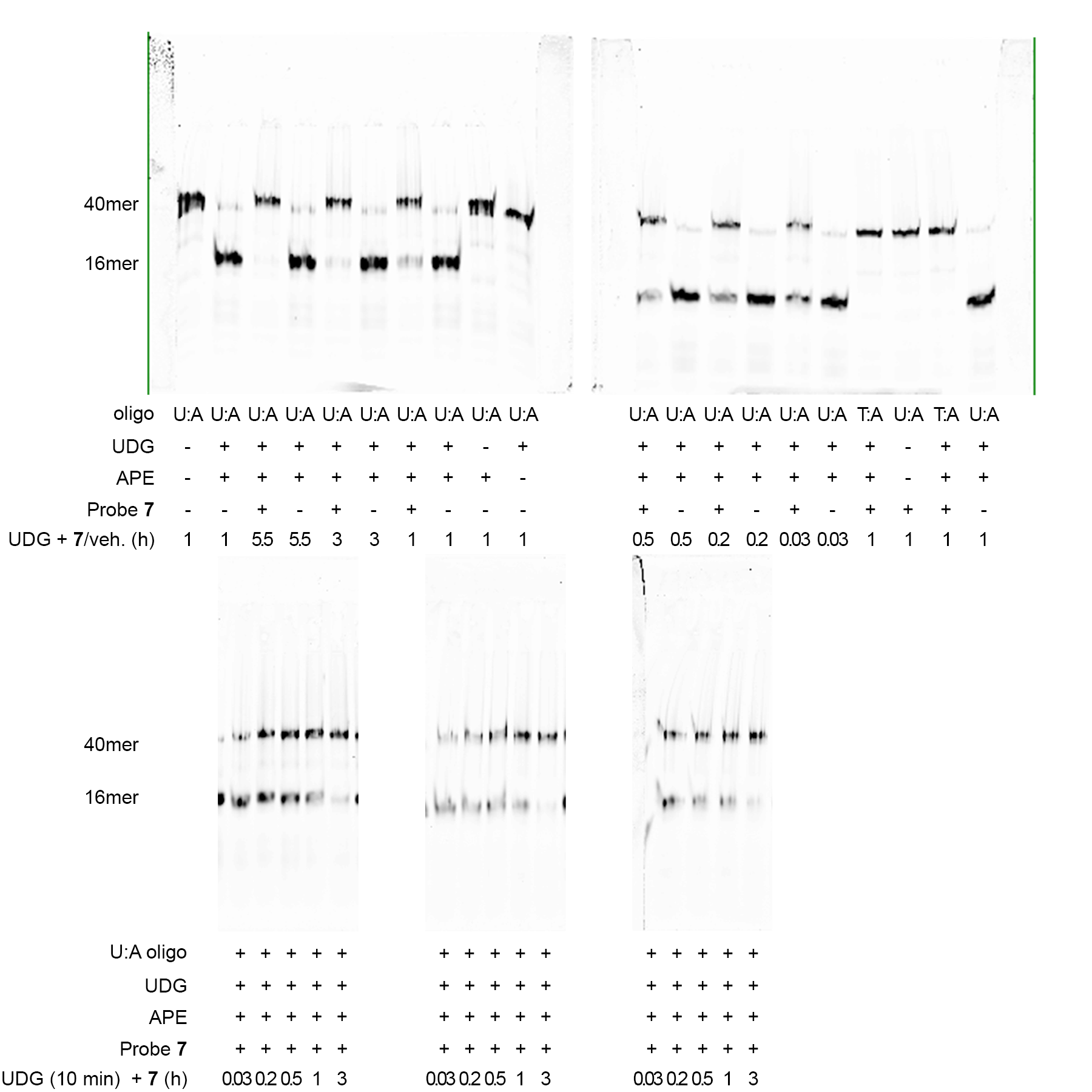


Figure S 5. Raw DNA denaturing PAGE (shown in grayscale) data used in quantification of HEX-labeled DNA in Figure 5. Lower panel shows three separate gels with adjacent bands (from an unrelated experiment) removed for clarity.

## Gel electrophoresis for Figure 6.


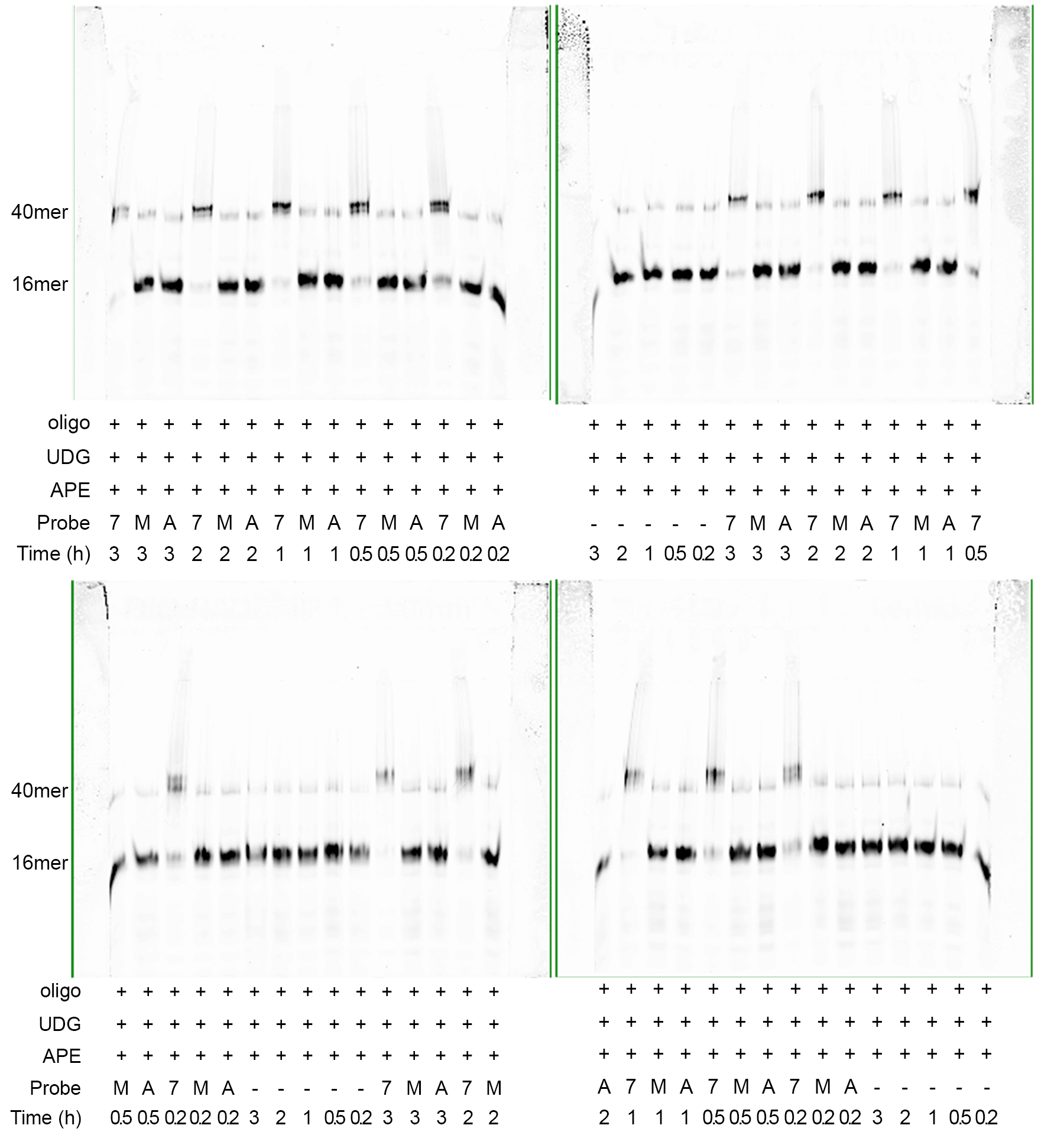


Figure S 6. Raw DNA denaturing PAGE data used in quantification of Figure 6A.


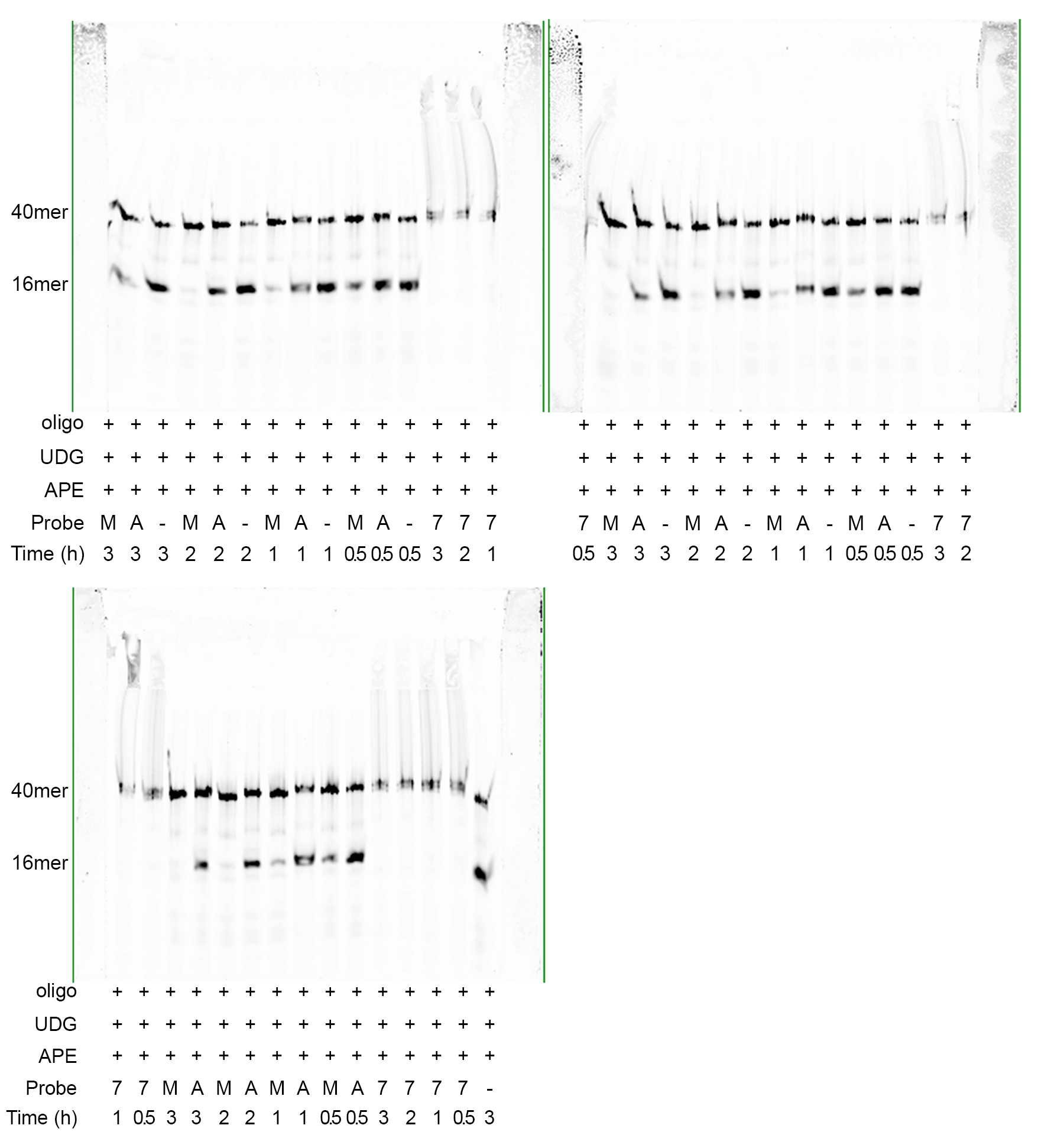


Figure S 7. Raw DNA denaturing PAGE data used in quantification of Figure 6B.

## Western blot and q-RT-PCR analysis for Figure 11.


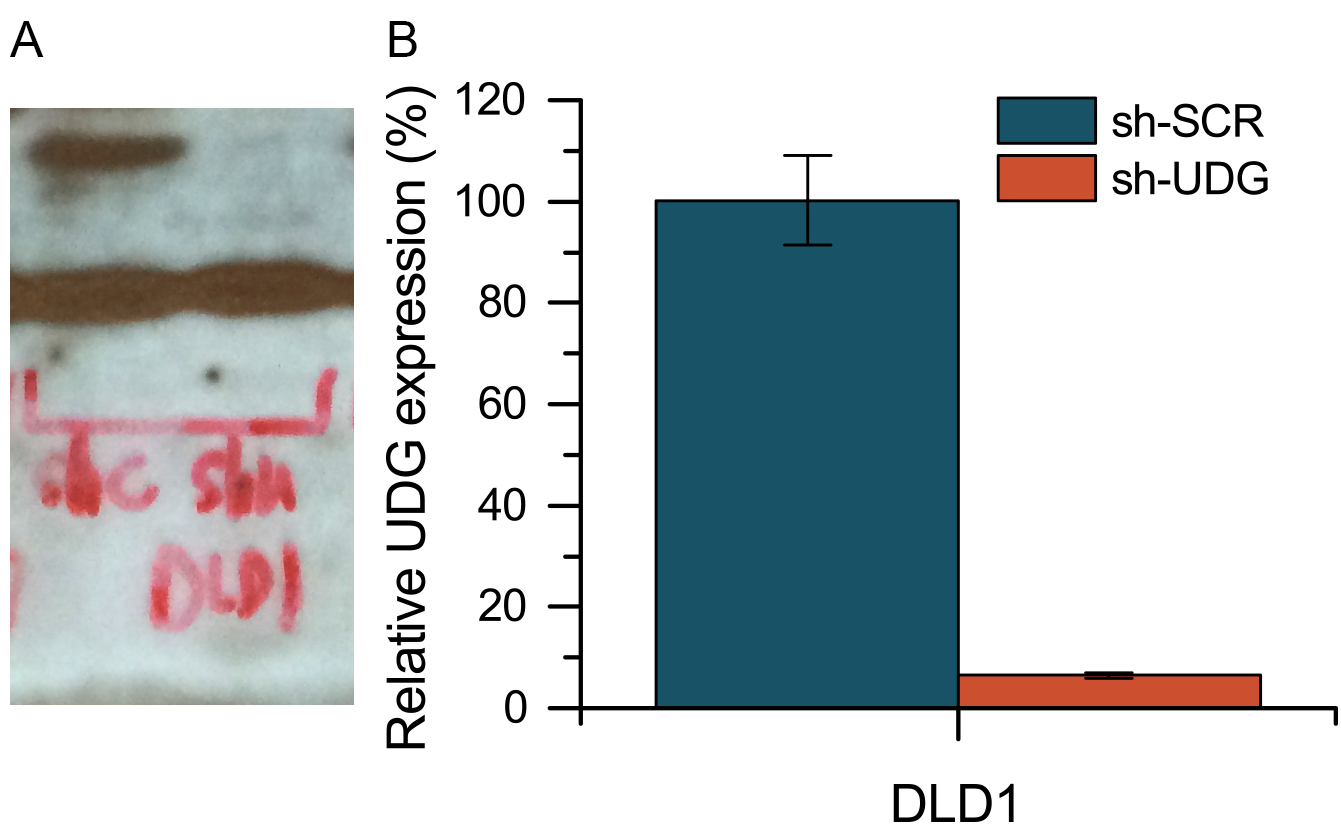


Figure S 8. Validation of shRNA-mediated UDG silencing. DLD1 cell line was transfected with either UDG targeted shRNA (sh-UDG) or scrambled control shRNA (sh-SCR). (A) UDG protein level was evaluated by western blots. (B) *UDG* mRNA expression was analyzed by q-RT PCR, normalized to β–tubulin, and expressed as a percentage compared with sh-SCR-transfected control.
